# Selection of sponge-associated bacteria with high potential for the production of antibacterial compounds

**DOI:** 10.1038/s41598-020-76256-2

**Published:** 2020-11-12

**Authors:** Riyanti, Walter Balansa, Yang Liu, Abha Sharma, Sanja Mihajlovic, Christoph Hartwig, Benedikt Leis, Frets Jonas Rieuwpassa, Frans Gruber Ijong, Heike Wägele, Gabriele M. König, Till F. Schäberle

**Affiliations:** 1 Institute for Insect Biotechnology, Justus-Liebig-University of Giessen, 35392 Giessen, Germany; 2 Faculty of Fisheries and Marine Science, Jenderal Soedirman University, 53122 Purwokerto, Indonesia; 3 Department of Fisheries and Marine Science, Politeknik Negeri Nusa Utara, 95821 North Sulawesi, Indonesia; 4 Fraunhofer Institute for Molecular Biology and Applied Ecology (IME), Branch for Bioresources, 35392 Giessen, Germany; 5 Centre of Molecular Biodiversity, Zoological Research Museum Alexander Koenig, 53113 Bonn, Germany; 6 Institute for Pharmaceutical Biology, University of Bonn, 53115 Bonn, Germany; 7 German Center for Infection Research (DZIF), Partner Site Giessen-Marburg-Langen, Giessen, Germany

**Keywords:** Biochemistry, Chemical biology, Drug discovery

## Abstract

The potential of sponge-associated bacteria for the biosynthesis of natural products with antibacterial activity was evaluated. In a preliminary screening 108 of 835 axenic isolates showed antibacterial activity. Active isolates were identified by 16S rRNA gene sequencing and selection of the most promising strains was done in a championship like approach, which can be done in every lab and field station without expensive equipment. In a competition assay, strains that inhibited most of the other strains were selected. In a second round, the strongest competitors from each host sponge competed against each other. To rule out that the best competitors selected in that way represent similar strains with the same metabolic profile, BOX PCR experiments were performed, and extracts of these strains were analysed using metabolic fingerprinting. This proved that the strains are different and have various metabolic profiles, even though belonging to the same genus, *i.e. Bacillus*. Furthermore, it was shown that co-culture experiments triggered the production of compounds with antibiotic activity, *i.e.* surfactins and macrolactin A. Since many members of the genus *Bacillus* possess the genetic equipment for the biosynthesis of these compounds, a potential synergism was analysed, showing synergistic effects between C14-surfactin and macrolactin A against methicillin-resistant *Staphylococcus aureus* (MRSA).

## Introduction

The oceans house highly dynamic and diverse microbiological habitats with an inherent high potential to discover species that are completely new, unique and highly adapted^1^. Furthermore, it can be assumed that this diversity represents a driving force for chemical novelty, opening up the chance to identify novel natural products with biological and pharmaceutical activities from this still underexplored part of the world^2,3^. Marine invertebrates, e.g. sea slugs (marine Heterobranchia), tunicates (Chordata) and sponges (Porifera), are a well-known bioresource for natural products^4–10^, which have the potential to advance into lead structures for drug discovery and development. Due to the fact that natural product research on sponges has been performed for many decades, various compounds with biological effects had already been discovered. For several of these compounds, evidence exists that the molecule of interest isolated from these sponges is actually produced by (endo)-symbiotic or otherwise associated bacteria, leading to the assumption that sponge-associated bacteria produce a multitude of novel bioactive molecules^11^. The host sponges and their bacterial microbiome constitute a mutualistic symbiosis, in which sponges provide space for the bacteria and the bacteria offer metabolites, e.g. nutrients of nitrogen and carbon fixation and specialized metabolites^12,13^. Furthermore, bacteria are involved in the chemical defence of the sponge by providing bioactive molecules^5,12–14^. Although the detailed mechanisms involved in shaping a sponge microbiome are unknown, it is clear that being able to influence potential competitors or predators represents an advantage for the producing microorganism(s), as well as for the host^13^. However, the interaction between different bacteria within the host is not known. Therefore, it is highly interesting to test and use the antibiotic effect of molecules that inhibit other bacteria also for pharmaceutical applications.

In recent years, antimicrobial resistance (AMR) has become a severe threat to global health systems. The antibiotic development pipeline is drying out and the number of antibiotic resistant bacteria is steadily increasing^15–18^. There are projections that by 2050 around 10 million people will die from antibiotic resistant infections per year^19^. Hence, novel targets and novel antibiotics represent an unmet medical need.

To fill the antibiotic development pipeline from the very beginning, natural products are still a highly promising resource. In recent years, it has been shown that there is still potential in the fraction of culturable bacteria for the identification of novel antibiotic compounds. In bioprospecting projects, hundreds of bacterial strains can easily be isolated, and approximately 10% of them possess antibacterial activities in preliminary screens. For example, Kuo et al.^20^ reported 15% of bacterial isolates from the marine sponge *Theonella swinhoei* to show antibacterial activity. From the marine sponges *Isodictya compressa* and *Higginsia bidentifera* 415 isolates were retrieved and 35 of them (8.4%) showed antibacterial activity^21^. Using the marine sea slugs *Chromodoris annae*, *Chromodoris dianae*, *Chromodoris* sp. 30, *Chromodoris willani*, *Doriprismatica stellata*, *Hexabranchus sanguineus* egg mass and *Phyllidiella cf pustulosa* as bioresource, out of 49 isolated bacteria, 35 demonstrated antibiotic activity^22^. In addition, even well-investigated habitats and species proved themselves as most valuable, as shown by the identification of teixobactin from a bacterium isolated from a soil sample^23^, and of darobactin from *Photorhabdus* species^24^.

If it can be accepted that the potential for finding novel antibiotics is still high, it is of importance to select the most promising strains for further investigations. The technological development in the last years offers great tools for the dereplication of compounds and activities. Mass-based analyses using sensitive mass spectrometers in combination with molecular networking, or even genome sequencing to get insights into the metabolic potential of a strain will be applied in the future^25^. However, such research and development pipelines are costly, need highly trained researchers and are not accessible to every lab. Therefore, in this study, we tested if a classical microbiology-based approach could be suitable to perform first prioritization without the need of expensive state-of-the-art laboratories. This is of special interest as bioresources like sponges are very often accessible in regions with poor biotech infrastructure. We report here about our bioprospecting project, in which 835 marine bacterial isolates were retrieved from 10 different sponges and 108 of these showed antibacterial activity in a preliminary screen. The prioritization of these active strains, which were identified by 16S rRNA gene sequencing, was done using a competition assay. In a championship like assay, the isolates were challenged with each other. The strongest competitors, *i.e.* isolates that won most of the matches against other isolates, were selected for further analysis. In that way, 25 isolates were prioritized and analysed further. To validate if only identical isolates were selected by this approach and the strong inhibitory effect was based on the same compound, BOX PCR and chemical fingerprinting was performed. This revealed that different bacteria made it to the top places. Furthermore, antibacterial active molecules were isolated and positively tested for their synergistic effects against clinically relevant methicillin-resistant *Staphylococcus aureus* (MRSA).

## Materials and methods

### Sponge collection and bacteria isolation

Sponges were collected by scuba diving in Enepahembang East Tahuna, Sangihe Islands, North Sulawesi Indonesia at a depth of ~ 1–9 m during July 2017 at the geographical position around 3°36′00.7″ N, 125°29′44.5″ E. Details are described in SI.

### Primary screening for antibacterial activity

The isolated marine bacteria were cultivated on appropriate solid medium at 30 °C for 1–7 days, depending on the growth rate of the respective strain. Then, an agar-plug (diameter 6 mm) was punched out and transferred to the test plate. On the latter, cultures of *Escherichia coli* K12 or *Micrococcus luteus* ATCC 4698 were swabbed on to the surface of Luria–Bertani agar (tryptone (10 g/L), yeast extract (5 g/L), NaCl (10 g/L), agar (20 g/L)) before plug assembly. The test plates were incubated at 30 °C (24–48 h, depending on the growth of the screening strain) and the inhibition zones were measured.

### Identification of active strains

The active isolates were identified by 16S rRNA gene sequencing. Genomic DNA of the isolates was extracted using the genomic DNA kit (Analytik Jena). PCR amplification was carried out using the primer pair pA (5′-AGAGTTTGATCCTGGCTCAG-3′) and pH (5′- AAGGAGGTGATCCAGCCGCA-3′). The PCR was performed in a total volume of 40 µL including 2 µL of DNA template, 2 U of Taq Polymerase (Promega, Madison, USA), 1× green buffer, 0.2 mM dNTPs, 0.5 µM primer pA, 0.5 µM primer pH, 1.25 mM MgCl_2_ and 5% of dimethyl sulphoxide (DMSO). PCR was performed in a Biometra TRIO Thermal Cycler (Analytik Jena) using the amplification conditions as follows: 95 °C 5 min (denaturation), 50 °C 45 s (annealing), 72 °C 1 min (elongation), and 72 °C 5 min (final elongation). Amplified 16S rRNA gene fragments were purified using the Promega SV Gel and PCR Clean Up System kit. Purified PCR products were sequenced (Eurofins Genomics). The obtained forward and reverse sequences were assembled. Then, a BLAST analysis was done to identify closest homologues. Sequences with > 98% sequence similarity (over an average of 1372–1400 bases) to their closest phylogenetic neighbour were assigned to the species level. Sequences with ˂ 98% sequence similarity were identified to the genus level^26^. The 16S rRNA gene sequences of the isolates have been deposited in GenBank with the accession numbers MT314037-MT314061 (Supplementary Table S1). Phylogenetic tree based on the 16*S* rRNA gene was constructed in MEGA X by using the Maximum Likelihood method (default settings, 1000 bootstraps) based on the Tamura 3-parameter model. The percentage of trees in which the associated taxa clustered together is shown next to the branches. Initial tree(s) for the heuristic search were obtained automatically by applying Neighbor-Joining and BioNJ algorithms to a matrix of pairwise distances estimated using the Maximum Composite Likelihood (MCL) approach^27^.

### BOX PCR

#### Preparation of samples and PCR conditions

The bacterial strains were grown on ISP2 agar plate at 30 °C overnight. Several bacterial colonies were transferred into the collection micro tubes (Cat.No.19560, Qiagen) with 2 Zirconia beads (2.3 mm Carl Roth, Art.: N036.1) and dissolved in 200 µL of sterile water. The mechanical cell disruption was performed by TissueLyser II for 2 × 1 min at 30 Hz. The samples were centrifuged for 2 min at 3220×*g*. One microliter of supernatant was used as a DNA template for the repetitive element palindromic-polymerase chain reaction (rep-PCR) analysis with BOXA1R oligonucleotide (CTACGGCAAGGCGACGCTGACG)^28^. The rep-PCR was carried out in a total volume of 25 µL including 1 µL of DNA template, 0.625 U of Dream Taq Polymerase, 1X Dream Taq buffer, 200 µM each dNTP, 1 µM primer and 0.4 µg/µL BSA. The following PCR conditions were used: 95 °C for 3 min followed by 30 cycles of 94 °C for 30 s, 53 °C for 1 min and 70 °C for 8 min, and a single final extension step at 70 °C for 16 min. The PCR products were analyzed by Lab Chip GX Touch HT microfluidics technology using 5 K DNA Assay (Cat.No. CLS760675, PerkinElmer, Inc.).

#### Statistical analysis/ Rep-PCR DNA fingerprint analysis

Gel image was normalized, bands were identified and data were statistically analysed by using GelCompare II software version 6.5 (Applied Maths, Belgium). The positions of bands on the gel were normalized using the DNA 5 K ladder (PerkinElmer, Inc.) from 100 to 7000 bp as an external reference standard. Similarity coefficient was calculated by band-based method of Dice, while unweighted pair group method with arithmetic averages (UPGMA) was used for cluster analysis.

### Competition assay

Initially, all strains to be tested were cultured on agar and in liquid medium 1–7 days. Liquid cultures represented the pre-culture for the test strain. Therefore, cells were separated by centrifugation at 6010×*g*, 5 min, 20 °C; then, inoculated into soft agar medium (10% agar), which was used to overlay the test plates. The tested bacteria were also inoculated and incubated until growth was visible on separate plates. The competition assay started afterwards; therefore, agar-plugs were prepared from the solid medium cultures and placed on the test plates.

### Screening culture extracts for antibacterial activity

The five most promising strains from the competition assay were fermented in ten different liquid media: ISP2, ISP2 + NaCl, LB, LB with ASW, marine broth, malt yeast extract, nutrient broth, starch nitrate with ASW, tryptic soy broth (TSB), tryptone and yeast extract medium with ASW. TSB medium consists of casein peptone (17 g/L), K_2_HPO_4_ (2.5 g/L), glucose (2.5 g/L), NaCl (5 g/L), soya peptone (3 g/L); tryptone and yeast extract medium with ASW consists of casein peptone (4 g/L) and yeast extract (2.5 g/L); the other media compositions are given in SI. Strains were inoculated from cryoculture into a preculture (20 mL in 50 mL flask), which was incubated at 140 rpm at 30 °C overnight. Then, 1 mL of this preculture was transferred into the main culture (100 mL new medium in 300 mL flask). Fermentation was done at 140 rpm, 30 °C for 2 days.

The fermented broths were extracted once using ethyl acetate (1:1). The resulting organic layer was collected and evaporated to dryness under reduced pressure using a rotary evaporator. Therefrom resulting organic extracts were used for antibacterial testing using the agar diffusion method. Extracts were dissolved in methanol (final concentration of 10 mg/mL) and 10 µL were applied onto a 6 mm paper disc that was allowed to dry at room temperature. Plates were pre-incubated for 1 h at 4 °C and subsequently transferred to 30 °C for 24–48 h. Methanol and carbenicillin were used as negative and positive control, respectively.

### MS measurements and molecular networking

To compare the various extracts and to dereplicate the compounds therein, molecular networking was carried out. The sixty-crude extract samples were dissolved in MeOH at the final concentration of 10 mg/mL and subjected to LC-HRMS measurement. Mass spectra were detected on a micrOTOF-QII mass spectrometer (Bruker, Billerica, MA, USA) with ESI-source combined with a HPLC Dionex Ultimate 3000 (Thermo Scientific, Darmstadt, Germany) utilizing an EC10/2 Nucleoshell C18 2.7 μm column (Macherey–Nagel, Düren, Germany) at 25 °C. MS data were obtained in positive mode over a range from 100 to 1000 *m/z*. For all ions above a threshold of 100, auto MS/MS fragmentation was performed with increasing collision energy (35–50 kV over a gradient from 500 to 2000 *m/z*) at a frequency of 4 Hz. The injection volume was 2 µL with a concentration of 1 mg/mL.

MS/MS data were converted from MassHunter data files (.d) to mzXML file format using MS Convert. The data were uploaded to the Global Natural Products Social (GNPS) molecular networking (https://gnps.ucsd.edu/). Network files were visualized using the program Cytoscape 3.7.2. Dereplication was done by comparing the MS^2^ spectra with the reference spectra in GNPS spectral libraries^25^.

### Metabolic fingerprinting

Bacteria were inoculated from cryoculture in ISP2 liquid medium supplemented with 2% NaCl and incubated overnight at 30 °C and 140 rpm. Then, ISP2 agar plates (2 per strain) were inoculated each with 50 µL of preculture and incubated at 30 °C for 2 days. The agar plates were cut into small pieces, macerated with ethyl acetate and placed on a shaker overnight. The solution was filtered and the resulting organic extract was dried in a rotary evaporator under vacuum. The concentration of this crude extract was adjusted to 50 mg/mL and subjected to LC-HRMS measurement (procedure done in triplicates).

MS-analysis was performed on 1290 UHPLC system (Agilent, Santa Clara, CA, USA) equipped with DAD, ELSD and maXis II (Bruker, Billerica, MA, USA) ESI-qTOF-UHRMS with the following gradient: A = H_2_O, 0.1% formic acid (FA); B = acetonitrile, 0.1% FA; Flow: 600 µL/min; 0 min: 95% A; 0.30 min: 95% A; 18.00 min: 4.75% A; 18.10 min: 0% A; 22.50 min: 0% A; 22.60 min: 95% A; 25.00 min: 95% A. Column oven: 45 °C. Column: Acquity UPLC BEH C18 1.7 µm (2.1 × 100 mm) with Acquity UPLC BEH C18 1.7 µm VanGuard Pre-Column (2.1 × 5 mm).

Data processing was performed with DataAnalysis 4.4 (Bruker, Billerica, MA, USA) using sodium formate for recalibration, RecalculateLinespectra (threshold 10,000) and FindMolecularFeatures (0.5–25 min, S/N = 0). Bucketing was performed using ProfileAnalysis 2.3 (Bruker, Billerica, MA, USA) (30–1080 s, 100–1600 *m/z*, Advanced Bucketing with 24 s 5 ppm, no transformation, Bucketing basis = H+). The bucket table was subsequently used as input for analysis via R. R (version 3.6.0)^29^ with libraries readr^30^, coop^31^, gplots^32^, data.table^33^, parallelDist^34^ and devtools^35^ were used. For heatmap-generation with several sidebars a variation of heatmap.2 by Griffith^36^ was used. Hierarchical clustering in the heatmap is performed with function “hclust” based on “complete linkage” of the cosine similarity results. The complete R-script is deposited here: Cosine-V2.R GitHub repository https://github.com/christoph-hartwig-ime-br/cosine-V2; https://dx.doi.org/10.5281/zenodo.3932968. For sample comparison, the cosine similarities (dot product of vectors) between samples were calculated. Samples were sorted according to clustering results and pairwise similarities were used to determine metabolic groups. If the pairwise similarity between to subsequent clustered samples is at the threshold (e.g. 0.9) or higher, they belong to the same metabolic group. Cosine similarity data was also analyzed inside of triplicates and between strains. For outlier in triplicates MS-data were inspected. If the differences could be explained due to large concentration differences, outliers were included in the same metabolic group as the rest of the strain.

### Co-cultivation

Selected bacteria (one representative of closely related strains of the 108 active ones with a different species) were cultured in liquid media in a co-cultivation approach. Bacterial strain 1 and bacterial strain 2 were inoculated separately in a 150 mL Erlenmeyer flask as pre-culture. Then, the experiment was conducted as follows: (a) Strain 1 was inoculated as single strain control, (b) strain 2 was inoculated as single strain control, (c) strain 1 was inoculated first and strain 2 was inoculated after 1 day in the same flask, (d) strain 2 was inoculated first and strain 1 was inoculated after 1 day in the same flask, (f) strain 1 and strain 2 were inoculated at the same day in the same flask, (e) medium was used as negative control.

The activity was measured based on the zone of clearance around the paper discs. The co-cultivation experiments that showed the highest activities were selected to perform the large scale cultivation: (1) *Bacillus* sp. EP6-817 and *Lysinibacillus sphaericus* EP6-121 inoculated at the different day in NB medium (Peptone 5 g/L, Malt extract 3 g/L, NaCl 5 g/L) and cocultivated for 1 day at 30 °C with shaking at 140 rpm; (2) *Verrucosispora* sp. EP6-325 and *Bacillus* sp. EP6-454 inoculated at the same day in M1 medium (Starch 10 g/L, Yeast extract 4 g/L, Peptone 2 g/L) and cultivated for 3 days at 30 °C with shaking at 140 rpm.

### Fractionation and purification

Cultures (12 L) were extracted with ethyl acetate by liquid–liquid separation (1:1). The generated crude extract (4.6 g) from the co-cultivation of *Bacillus* sp. EP6-817 and *Lysinibacillus sphaericus* EP6-121 was first submitted onto a reverse phase silica C-18 column (Puriflash C18-HP 30 μm F0080 Flash column). The gradient was increased from 10% MeOH to 100% MeOH in 40 min, then kept at 100% MeOH for 10 min with flowrate of 25 mL/min. This yielded 4 fractions which were collected according the UV absorption of the substance. By bioassay-guided isolation, fraction 4 was purified by Sephadex LH 20 (mobile phase 100% MeOH) to yield one active fraction (32.4 mg) which was further purified using a Shimadzu HPLC (Shimadzu Deutschland GmbH, Duisburg, Germany) with a reverse phase column (column EC 250/4.6 Nucleodur C18 Gravity-SB, 5 μm) at a flowrate of 1 mL/min. Gradient increased from 75% MeOH to 90% MeOH in 25 min. One active compound was identified as macrolactin A (5.2 mg) according to the 1D and 2D NMR spectra as well as the LC-HRMS spectra (Figs. S1-S6). The crude extract from the co-cultivation of *Verrucosispora* sp. EP6-325 and *Bacillus* sp. EP6-454 generated 6 fractions via Sephadex LH 20 (mobile phase 100% MeOH). Then, the active fraction 1 was loaded on a reverse phase silica C-18 (Puriflash C18-HP 30 μm F0040 Flash column; gradient increased from 50% MeOH to 90% MeOH in 20 min) to yield 2 active subfractions. Subfractions 1 and 2 were further purified by semi-prep HPLC (subfraction 1: gradient increased from 80% MeOH to 95% MeOH in 30 min; subfraction 2: gradient increased from 85% MeOH to 100% MeOH in 38 min) to isolate four compounds. C14-surfactin (7.2 mg) and C15-surfactin (4.3 mg) were identified by 1D NMR spectra together with LC-HRMS (Figs. S7-S9), while C16-surfactin (1.2 mg) and C17-surfactin (1.5 mg) were identified by LC-HRMS (Fig. S10).

### NMR experiments

NMR spectra (1D and 2D) of isolated compounds were recorded in CD_3_OD (Aldrich, St. Louis, MO, USA) using a Bruker Avance II 400 MHz NMR spectrometers (Bruker, Ettlingen, Germany).

### Minimal inhibitory concentration and synergistic effect of surfactin and macrolactin

The minimal inhibitory concentration (MIC) and fractional inhibitory concentration (FIC) were determined as previously reported^37^. In brief, a panel of Gram-negative *E. coli* ATCC 25922 (wild-type and Δ*tolC* mutant), Gram-positive *B. subtilis* DSM 10, *S. aureus* ATCC 25923 (methicillin-sensitive), and *S. aureus* ATCC 33592 (methicillin-resistant, MRSA) were grown in cation-adjusted Mueller–Hinton II broth (CAMHB, Becton Dickinson) over-night, *Listeria monocytogenes* DSM 20600 was grown in brain heart infusion (BHI) medium supplemented with 1% (v/v) Tween 80 over 2 days of incubation at 37 °C, until the 30 mL preculture was turbid. Next, the preculture was adjusted to McFarland 0.5–1 turbidity standard (approx. 1.5 – 3.0 × 10^8^ cfu per mL) and diluted 1:600 in fresh media as seeding cell suspension for the growth inhibition assay. Purified macrolactin A and surfactins were dissolved in DMSO (12.8 mg/mL final concentration), spotted onto 96 well plates and 1:2 dilution series in a volume of 100 µL of the seeding cell suspensions were prepared on 96 well plates to obtain a final compound concentration of 128 – 0.0625 µg/mL. Growth and sterility controls were added (DMSO as solvent control), and gentamycin served as positive control. Endpoint MIC values were measured after 18 ± 2 h at 180 rpm shaking speed and 37 °C at 85% relative humidity. Readout was obtained by turbidity absorption measurement at 600 nm and chemiluminescence-based ATP-quantification using BacTiter Glo Microbial Cell Viability Assay (Promega) according to the manufacturer’s recommendations on a LUMIstar Omega plate reader (BMG Labtech). MIC values were calculated as growth inhibition (I%) ≥ 80% from at least three independent measurements (n ≥ 3). Synergistic interaction of purified surfactin and macrolactin A was tested using the chequerboard assay in 96 well plate format^37^. In brief, fractional inhibitory concentration (FIC) values for macrolactin A and the surfactins C14 were calculated for each compound concentration combination using the equation FIC_cpd_ = Conc._cpd_ / MIC_cpd_. The fractional inhibitory concentration index (FICI) values were calculated as FICI = FIC_Macrolactin A_ + FIC_Surfactin_, whereas FICI ≤ 0.5 indicated synergistic effects between both compounds and FICI values > 4 antagonism.

## Results

### Sponge collection and bacteria isolation

During our continuous efforts to identify natural products from marine bioresources, 10 sponge specimens covering 10 different Demospongia species, were collected from Sangihe Island, Indonesia (a description of the specimens with a preliminary genus or species identification is provided in the Supplementary Table S2). From these samples, associated bacteria were isolated using classical agar plate-based methods with various media. In total, 835 marine bacteria were isolated as axenic culture. From most sponge samples, approximately 100 cultures were isolated. However, from two sponge samples, *i.e.* specimen EP10 (cf. *Aaptos suberitoides*) and EP15 (cf. *Agelas nakamurai)*, only about 20 isolates were retrieved in this study. Comparing the isolation efficiency of the different media, the highest number of isolates (143) was obtained from SNA medium (consisting of starch and salts); while from NB medium (complex medium) only 23 isolates were retrieved (Fig. S11).

### Screening for antibacterial activity

**Figure 1:**
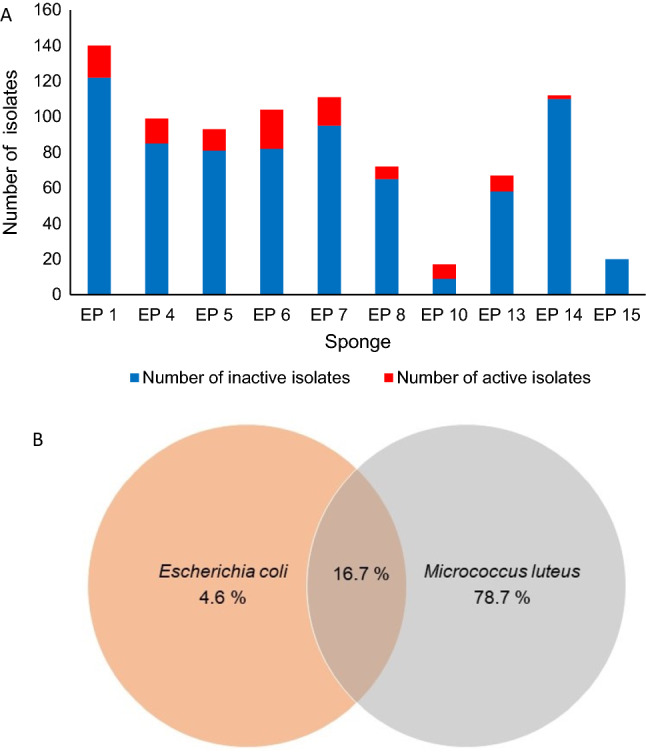
Number of active strains. (**A**) Bacterial isolates (active isolates in red) retrieved from each sponge specimen (for description of sponges see Table S2). **(B)** Percentage of the 108 isolated active bacteria that showed activity solely against Gram-positive *M. luteus*, Gram-negative *E. coli* and against both test strains.

Concerning a biological activity of the isolates, we focussed on antibacterial activity. The 835 isolates were screened for activity against the Gram-positive bacterium *M. luteus* ATCC 4698 and the Gram-negative bacterium *E. coli* K12 using a growth inhibition assay on agar plates. This primary screening revealed that 12.9% (108) of the isolates showed antimicrobial activity against at least one of the bacteria tested (Table S3). Among the 108 isolates active in the primary screening, 4.6% inhibited solely Gram-negative *E. coli* bacteria, 78.7% inhibited Gram-positive *M. luteus* and 16.7% inhibited both test strains (Fig. 1).

Thereby, the highest number of active isolates originated from the sponge EP6 (22 out of 104 isolates showed activity) and the lowest number from sponge EP14 (2 out of 112 isolates showed activity). From sponge sample EP15 no active isolate was retrieved. However, it must be considered that from this sponge only a very low number of bacteria was isolated in total (Fig. 1).

### Identification the active strains

In a next step, the active isolates were identified using 16*S* rRNA gene sequencing. Identified strains belonged to the phyla Firmicutes (76.9%), Proteobacteria (17.6%) and Actinobacteria (5.6%), respectively. On genus level, 12 genera were present, whereby two third of the strains belonged to *Bacillus* (66.7%), followed by *Pseudomonas* (6.5%), *Staphylococcus* (5.6%), *Lysinibacillus* (4.6%), and *Solwaraspora* (3.7%). The other seven genera had a share below 3% (Table 1).

**Table 1:** Phyla and genera of the active bacterial strains based on 16*S* rRNA gene sequencing.

Phyla	Genus	Occurrence (%)
Firmicutes	*Bacillus*	66.67
Proteobacteria	*Pseudomonas*	6.48
Firmicutes	*Staphylococcus*	5.56
Firmicutes	*Lysinibacillus*	4.63
Actinobacteria	*Solwaraspora*	3.70
Proteobacteria	*Citrobacter*	2.78
Proteobacteria	*Enterobacter*	2.78
Proteobacteria	*Serratia*	2.78
Proteobacteria	*Cronobacter*	1.85
Proteobacteria	*Leclercia*	0.93
Actinobacteria	*Brevibacterium*	0.93
Actinobacteria	*Verrucosispora*	0.93

### Competition assay

An important step in natural product research is the prioritization of bacterial strains for further investigation. Therefore, a strong dereplication platform, mostly based on MS analyses of the extracts derived from the strains, is key. Another option is the in silico analysis of the strains following genome sequencing. However, both platforms are relatively costly and not available at all laboratories. The goal of this project was to test if a prioritization of strains is possible depending on agar plate-based competition assays. This methodology can be done by microbiologists in virtually every lab, also in remote areas without expensive instrumentation.

**Figure 2:**
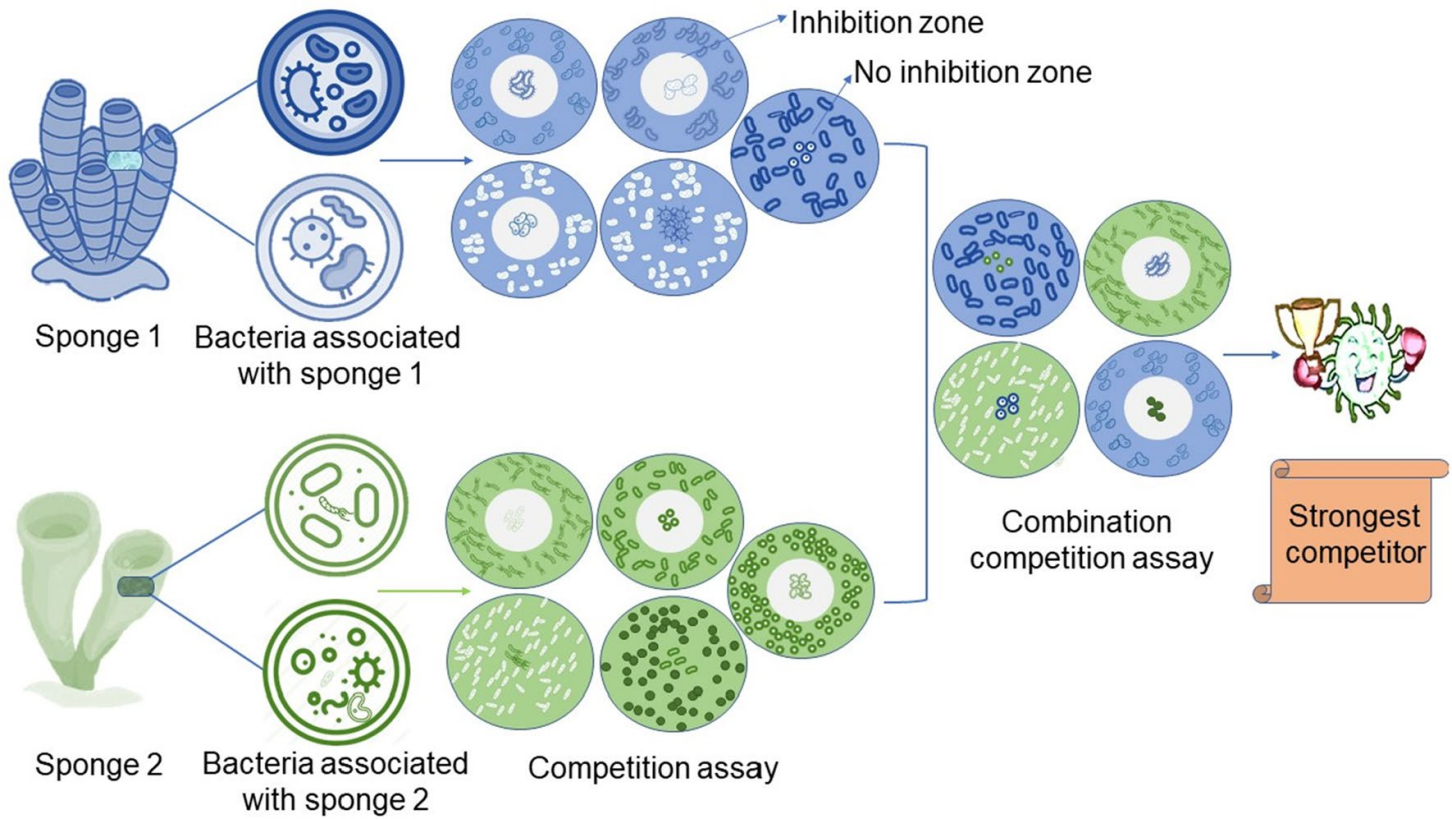
Scheme of the competition assay. Bacteria were isolated from the sponge specimens. As example a blue and green coloured sponge are shown. All axenic cultures derived from one sponge were tested against all the others from one sponge. Therefore, bacteria were cultured and agar plugs were transferred to agar plates inoculated with the test strain. Best competitors (inhibiting most of the test strains, which was observed by inhibition zone) were selected and tested against best competitors derived from other sponges.

**Figure 3:**
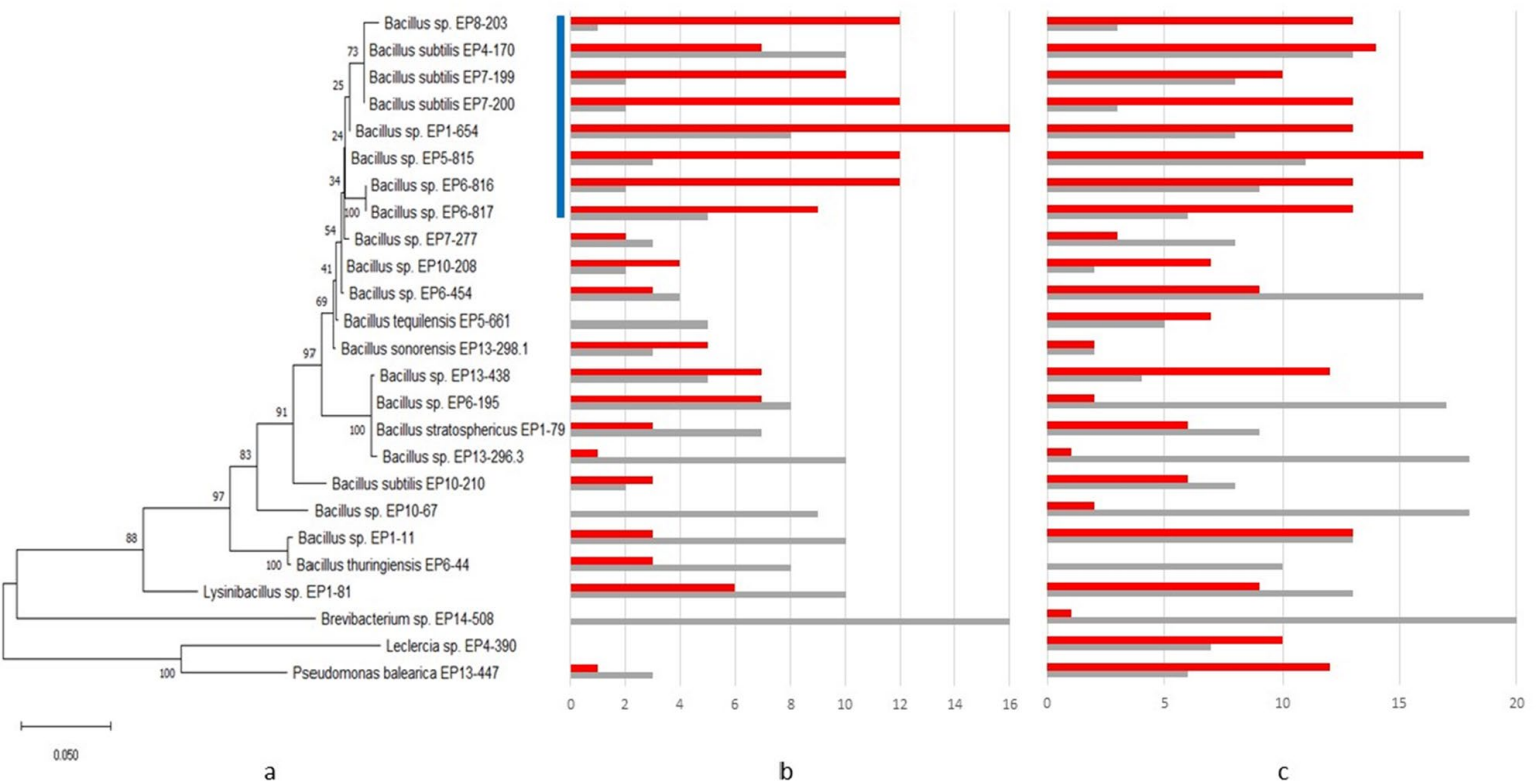
Phylogenetic tree and the activity/sensitivity profile of the 25 high competitor strains. (**a**) Phylogenetic tree based on 16S rRNA gene sequences. **(b)** Activity/sensitivity profile of the strains, which were cultivated in the medium they were originally isolated from. Therefore, different media were used. **(c)** All strains fermented in ISP2 medium supplemented with NaCl. The bar diagram indicates for each strain how many competitors were inhibited (red) and by how many itself was inhibited (grey). The blue bar indicates the branch of high competitors. Bootstrap values are given at the branches of the phylogenetic tree.

The underlying hypothesis is that the strongest competitors should be selected, since these strains have a higher probability of success for the identification of compounds with antibacterial activity. Strong competitors were regarded as the strains that inhibited most of the other strains originating from the same host sponge (Fig. 2). Hence, in a preliminary round, the strains were challenged in bilateral agar plate-based inhibition assays. Strains were incubated as axenic cultures on agar plates during 2–7 days depending on the growth rate of the respective strain. Then, plugs were punched out and transferred to a test plate inoculated with the test strain. After 24 h of incubation, the inhibition zone was measured. In that way, the strongest inhibitors (in total 25 strains) were selected and qualified themselves for the next round, i.e. the competition assay between the strains selected from the different sponge hosts. In the final round, the number of inhibited strains, inhibition zones and the number of strains inhibiting each strain were documented (Fig. 3 and Table S4).

*Bacillus* sp. EP1-654 was the winner of this challenge; since this strain inhibited 16 of the 24 other strains (67%). Thereby, it showed inhibitory activity against at least one bacterium from each host sponge, except the bacterial strain derived from sponge EP8, i.e. *Bacillus* sp. EP8-203. The latter strain was also a high competitor, like *Bacillus* sp. EP7-200, EP5-815 and EP6-816; it inhibited 12 out of 24 strains of the test set, respectively. Furthermore, *Bacillus* sp. EP8-203 showed the lowest sensitivity, since it was only inhibited by one competitor. *Brevibacterium* sp. EP14-508 was the weakest strain in the challenge. It did not inhibit one of the other strains and in turn was inhibited by 16 of the competitors (Fig. 3 and Table S4).

### Correlation of marine bacterial taxonomy and chemical fingerprint

**Figure 4:**
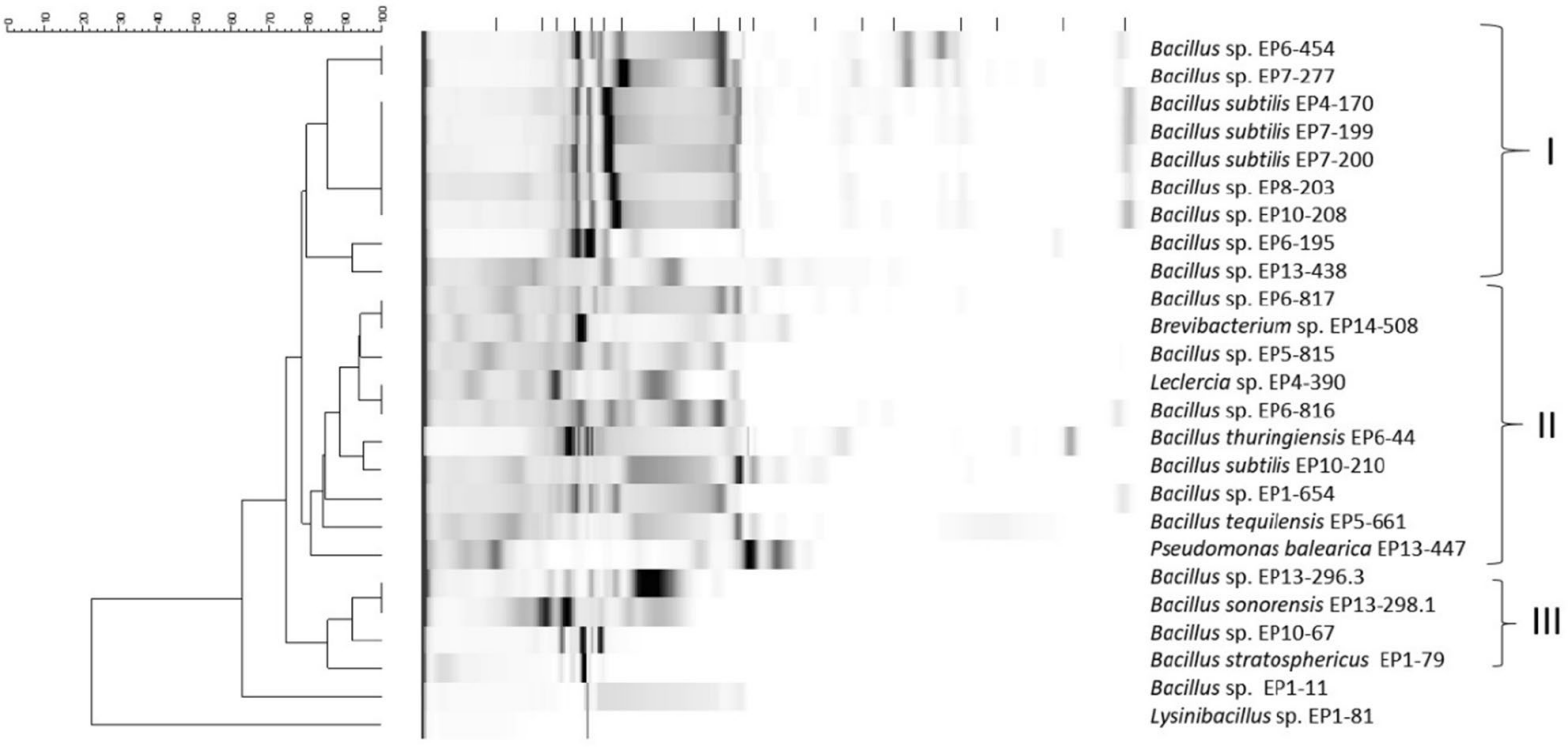
Dendrogram showing the relatedness of the isolated strains as determined by a PCR DNA fingerprint analysis with BOXA1R. Relationships were determined by using Dice similarity coefficient and UPGMA clustering method. The number gives the internal identifier of the respective strain; cluster groups with a similarity higher than 75% are indicated by brackets.

As mentioned before, the top five competitors belonged to the genus *Bacillus*, which was also the most abundant genus in this collection. To get an idea about the phylogenetic relationship and if other genera could also be found among the strong competitors, a phylogenetic tree based on the 16*S* rRNA gene sequences was built (Fig. 3). The activity and sensitivity pattern were plotted to this tree and showed that the highest competitors belonged to a branch of closely related strains. This trend was observed (i) when all strains were fermented in the same medium (which gives a better comparability) and (ii) when the strains were fermented in the medium they were originally isolated from. Since it is known that variation of the growth conditions, e.g. medium applied, results in a changed metabolome, the isolation medium was also used (Fig. 3). Since this could be an indication that the strains selected by the competition assay would be highly redundant, a BOX PCR was performed (Fig. 4).

A dendrogram based on BOXA1R fingerprint data was constructed by using Dice similarity coefficient and the UPMGA cluster analysis method to determine the phylogenetic relationship of the strains with higher resolution. This revealed that a few strains show a high similarity of the band pattern, but in general, a quite diverse pattern was observed. Taking into account the similarity higher than 75%, the strains are grouped in three major clusters (Fig. 4). The first group includes solely *Bacillus* strains. The average similarity coefficient among these strains was 93% while the strains EP4-170, EP7-199, EP7-200 and EP8-203 appeared to be identical. The second group represents the most heterogeneous group involving the species from different genera but with the average similarity of 90%. The third group includes four *Bacillus* strains isolated from EP 1, 10 and 13. They had the same similarity coefficient as group I.

**Figure 5:**
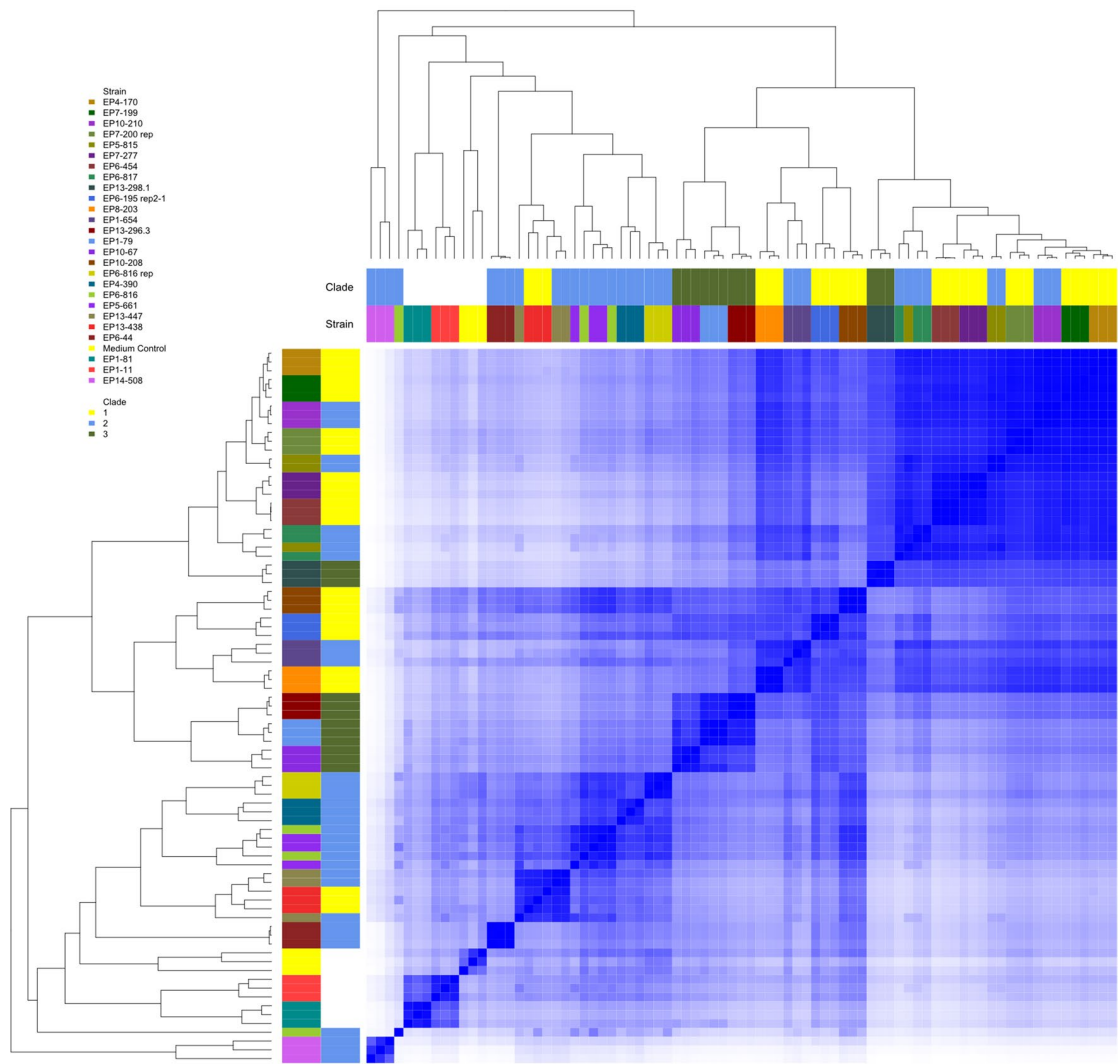
Cosine similarities of chemical fingerprints from 25 strains and medium control (n = 3 for all strains represented). Strain-IDs and clades (according to box-plot in Fig. 4) are color-coded in the sidebars. Clustering is based on cosine similarity profiles. (Further details in material and methods section).

In a next step, the 25 high competitors were subjected to a metabolomics analysis. The taxonomic similarity based on housekeeping genes does not necessarily reflect the similarity of strains in regard to their metabolome, since also closely related strains might carry different biosynthetic gene clusters (BGCs) encoding for antibacterial active natural products. Therefore, the crude extracts of these strains were analysed by chemical fingerprinting. The chemical fingerprint enables to judge the similarity of metabolomes, *e.g.* visualized in a dendrogram (Fig. 5, complete dataset is shown in Figure S13). Grouping of all extracts using a cosine similarity threshold of 0.9 resulted in 38 groups (cosine 0.8 in 19 groups). Analysis of cosine similarity between strain triplicates was performed and outliers were sorted to the rest of the triplicates if large differences in concentration led to the deviation while the Base Peak Chromatogram pattern was identical (Table S5, metabolic variations are shown in Figure S12). After inspection, 19 metabolic groups (excluding media controls) remained (Table S6, corresponding chromatograms Figure S12). While strain EP14-508 is most dis-similar to all other profiles (Fig. 5), it closely groups to other strains in the DNA analysis. The strains EP7-199 and EP7-200 showed even 100% identity in their 16S rRNA sequence; however, in the chemical fingerprint differences were detectable. This result matches the results of the BOX PCR, that already indicated slight differences, and of the competition assay, since in the latter strain EP7-200 inhibited more strains than EP7-199. Furthermore, the strains of metabolic group 1 are partly from different clades based on the DNA fingerprint (Fig. 4).

### Natural products produced

The strongest competitors belonged all to the genus *Bacillus*. The top five (*Bacillus* sp. EP7-200, EP8-203, EP1-654, EP5-815 and EP6-816) were selected and fermented in ten different liquid media to test the crude extracts for antibacterial activity against *E. coli* and *M. luteus*. Extracts of nearly all strains were active against both, Gram-positive and Gram-negative test bacteria. *Bacillus* sp. EP6-816 was the only exception, since the extract showed only activity against *M. luteus*. However, the results differed depending on the cultivation medium. For example, *Bacillus* sp. EP7-200 and EP8-203 displayed activity against Gram-negative and Gram-positive bacteria only, if grown on ISP2 medium. Instead, *Bacillus* sp. EP1-654 and EP5-815 were active against both tested bacteria after fermentation in MYE and NB medium. Furthermore, the results of the activity screening varied between solid and liquid cultivation. *Bacillus* sp. EP7-200 showed activity against both of the tested bacteria in the agar plug assay when cultivated on ISP2 medium supplemented with NaCl. However, this activity was not observed by testing the ethyl acetate extract obtained from liquid broth. Then, only activity was observed against *M. luteus* (Table S7).

In addition to the activity tests, the extracts from the five selected strains in ten media, as well as the medium controls were subjected to LC–MS/MS analysis. Thereby, some compounds could be dereplicated based on the GNPS natural products library, *e.g.* surfactin C12, surfactin C14, surfactin C15 and lichenysin A (Figs. S14, S15, S16).

The production of surfactins was a common feature for many of the here isolated bacteria when cultivated on solid medium. In liquid medium, it was observed that especially in co-culture experiments the production of this compound series was induced. The C14 to C17 surfactins were successfully isolated from a co-culture, in which *Verrucosispora* sp. EP6-325 and *Bacillus* sp. EP6-454 were inoculated at the same day in M1 medium (Figs. S7–S10).

**Figure 6:**
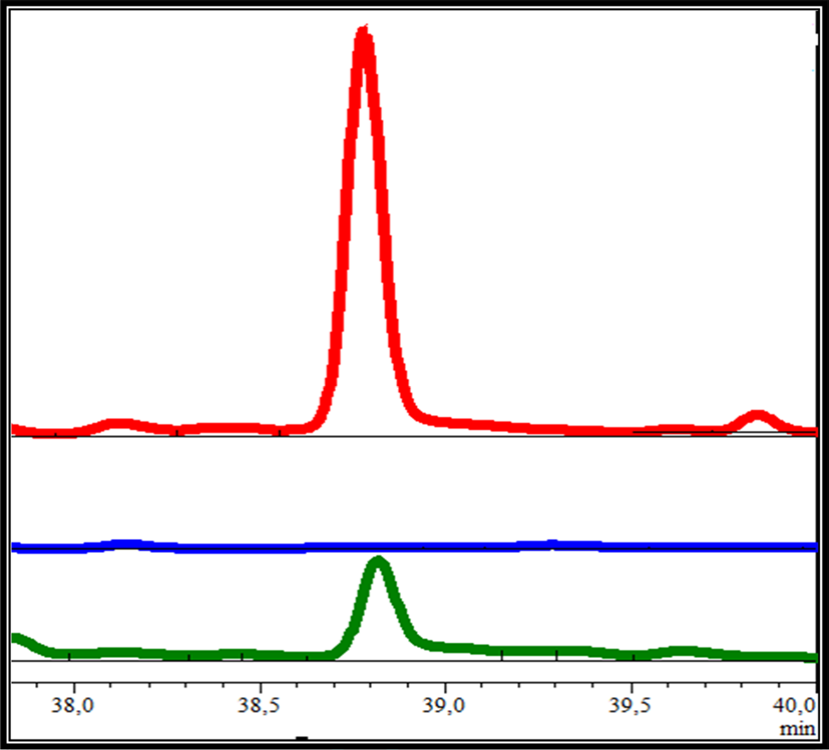
High performance liquid chromatography (HPLC) profiles of EtOAc extracts. The extract of pure cultures of *Bacillus* sp. EP6-817 is depicted in green, of *L. sphaericus* EP6-121 in blue, and of the co-culture in red. It can be seen that the production of macrolactin A is induced in the co-culture.

The experiments with axenic cultures do not reflect the conditions of the microbiome of a sponge in nature. Therefore, further co-culture experiments were performed, to challenge the strain with the presence of a competitor. A co-culture of *Bacillus* sp. EP6-817 and *Lysinibacillus sphaericus* EP6-121 was tested in a way that the one strain was incubated for one day before the other strain was added, as well as inoculation on the same day in one flask. A clear increase in the production of one compound was observed when *Bacillus* sp. EP6-817 was cultured for 1 day, before the culture was inoculated with *L. sphaericus* EP6-121 (Fig. 6, Fig. S17). This peak was not detected in *L. sphaericus* EP6-121 extracts and in much lower abundance if *Bacillus* sp. EP6-817 was cultivated alone. From the co-culture, this peak was purified and its structure elucidated using NMR experiments, proving it to be macrolactin A (*m/z* 425.2308 [M + Na]^+^, Figs. S1–S6).

### Synergistic effect of surfactin and macrolactin

**Figure 7:**
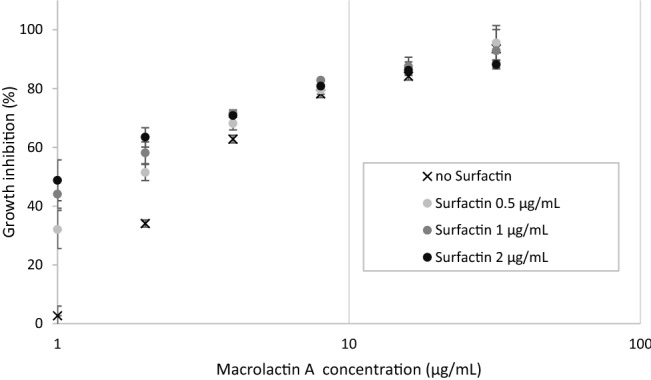
Synergistic combination effect of macrolactin A and C14 surfactin against *S. aureus* ATCC 33592 (methicillin-resistant MRSA strain). Dose–response of macrolactin A without surfactin (black cross) is compared with increasing sub-MIC concentrations of surfactin C14 (0.5–2 µg/mL surfactin, light grey to black dots). Data is depicted as average values ± standard deviations from four independent experiments, n = 4.

Surfactin and macrolactin appeared to be compounds of which the production is increased by the presence of a competitor strain. Furthermore, both corresponding BGCs can be detected in many sequenced *Bacillus* genomes. Therefore, their antibacterial effects were analysed as single compounds and in combination. First, the minimal inhibitory concentration (MIC) values of the single compounds were determined against a panel of bacteria in broth microdilution assays, *i.e.* Gram-negative *E. coli* ATCC 25922, *E. coli* ATCC 25922 ΔTolC and Gram-positive *B. subtilis* DSM 10, *S. aureus* ATCC 25923 (methicillin-sensitive), *S. aureus* ATCC 33592 (methicillin-resistant, MRSA), *L. monocytogenes* DSM 20600. However, none of the surfactins were active against these strains (MIC > 128 µg/mL). Instead, macrolactin A was active against the Gram-positive test strains, except *B. subtilis* DSM 10 (Table S8). In addition, combination effects between macrolactin A and surfactin C14 were assessed by chequerboard assays (Table S9), using *E. coli* ATCC 25922, *B. subtilis* DSM 10 and *S. aureus* ATCC 33592 (methicillin-resistant, MRSA) as test strains. In this experiment, the fractional inhibitory concentration indices (FICI) for each compound concentration and compound combination were calculated, whereas FICI values ≤ 0.5 indicated synergism for the combination of macrolactin A and surfactin C14. The presence of sub-MIC concentrations of surfactin C14 (as low as 0.5–2 µg/mL) in combination with macrolactin A (sub-MIC dosing between 1–2 µg/mL) showed a synergistic antimicrobial effect (FICI ≤ 0.5). As compared to macrolactin A without any supplement, the percentage of growth inhibition is enhanced by factor >10 at 1 µg/mL macrolactin A and surfactin dosing ≥ 0.5 µg/mL, whereas at 2 µg/mL macrolactin A the percentage of growth inhibition is doubled by surfactin (Fig. 7 and Fig. S18). At higher macrolactin A concentrations (≥ 4 µg/mL), the synergistic effect cannot be resolved anymore.

## Discussion

Sponge-associated bacteria represent an important bioresource for antibiotic compounds. In this study, 12.9% of isolated bacteria exhibited antimicrobial activity against at least one test organism. This number of active isolates is in the range reported for other bioprospecting projects, which was in the range of 2–34%^21,38,39^. *E.g.,* 8 of 400 isolates (2%) from the coastal marine sponges *Amphilectus fucorum* and *Eurypon major* revealed activity against *E. coli* and *B. subtilis*^40^*.* In our study, Firmicutes is the phylum with the highest share (77%) on antibacterial activity, thereby especially genus *Bacillus* contributing to this, which is consistent with other reports^41,42^. However, several of these projects, aiming to isolate bacteria with antibiotic activity started with a lower number of isolates and selected the most promising isolate. For example, from 92 bacteria isolated from red algae, 33% showed antibacterial activity. Of the ten selected isolates, seven were of the genus *Bacillus*^41^. A total of 158 isolates were isolated from a sponge, thereunder twelve were active isolates, five of them belonging to Firmicutes^42^. In general, *Bacillus* have to be regarded as proliferative producers of natural products with various biological effects. Together with their sporulation efficiency this can be regarded as a significant advantage for survival in different environments^43^. However, this is also providing a bias in isolation projects, since the spore-formers easily survive different sampling and storage procedures and are fast growers on many standard media. Based on metagenomic approaches, the most dominant groups of sponge holobionts belong to the phylum Proteobacteria^44–46^. Therefore, it is obviously hard to compare the cultured communities from marine sponges directly, since variations in media and culture conditions can have a huge impact on the isolation and cultivation success^42^. Furthermore, the great plate count anomaly was also reported for sponge-derived bacteria, *e.g.* less than 1% of bacteria observed by microscopic analysis in sponge tissues could be cultured using standard medium^47^, and only a low number of bacteria within a sponge grew under laboratory conditions^12^.

In this study, the fraction of isolated bacteria from different sponges varied. However, the low number of 20 isolates originating from sponge EP 15 (*Agelas nakamurai)* can be explained by the presence of agelasines in this sponge^48^. Agelasines are sponge-derived compounds that show antibacterial, as well as cytotoxic properties. Hence, it might be that either associated bacteria were killed during storage and processing of the samples due to the active compounds present, or that due to the presence of these effective compounds the sponge does not need dedicated antibiotic producers in its microbiome.

To evaluate the approach of prioritizing strains active in a preliminary screening, based on their ability to inhibit other isolates from the same holobiont, the metabolomic fingerprints were analysed. The method was reported to provide a high-resolution strain discrimination, as shown for a group of closely related Streptomycetes^49^. Here, experiments were performed in triplicates, which grouped together; however, a few triplicate samples did not cluster together; they rather formed a group with other strains. This might indicate that these strains, that cannot be clearly differentiated by chemical fingerprinting, are very similar in their metabolome. Furthermore, three strains (strain code EP4-170, EP7-199 and EP7-200) that are highly similar on their metabolic fingerprint showed high identity in the BOX PCR band pattern, and formed a monophyletic clade in the 16*S* rRNA gene-based phylogenetic tree. However, even these three strains, which are closely related to *Bacillus subtilis*, did not show an identical activity and sensitivity pattern in the competition assay. Therefore, it can be concluded that analysis of the activity and sensitivity pattern of a strain allows differentiation and therewith prioritization, even between highly similar strains. This result is consistent with another study^50^, which reported significant differences in bioactivity and chemical diversity between strains of the same species. It might even be that strains, which are identical on the genetic level, could show a different reaction in strain interactions. The background of different hosts could have resulted in different fine tuning of the regulation mechanisms that in turn lead to the production of different (levels of) metabolites^20^.

In addition, the activity/sensitivity pattern of the five strongest competitors (all taxonomically related *Bacillus* species) was different. These bacteria should be prioritized for further analysis, because they can inhibit most of the competitors. The strains were isolated from five different sponges, which might point toward the fact that in each microbiome a high competitor *Bacillus* strain is selected. It is obvious that isolates from different sponges could produce varying active compounds. At present, most of the activities observed cannot be directly linked to a corresponding compound. However, sponge-derived Firmicutes (particularly the genus *Bacillus*), are well known for the production of antimicrobial compounds^51^. In this project, the dereplication of the extracts resulted in the identification of ‘classic’ *Bacillus* compounds, *e.g.* C12, C14, C15 surfactins and lichenysin A. Based on the fact that genus *Bacillus* in general possess many BGCs, which reflects the potential to produce various secondary metabolites, it can be expected that in nature a cocktail of compounds is used to tackle competitors. In terms of biological fitness, the ability to produce relatively low (sub-MIC) amounts of single antimicrobial compounds in combination, while maintaining or even exceeding the growth inhibition effects against competing microorganisms is of major importance. Synergy of at least two different molecules with different mode of action is given, if the effect of the combination at low dosage is larger than the additive effect of both compounds alone. This principle has shaped natural-product producing bacteria in their specialized ecological niches, whereas the classic medicinal approach is currently antibiotic treatment with one specific drug. In a historical retrospective, this has been a dead-end strategy that has assisted the emergence of antibiotic resistance, reducing effective treatment options in the future. The analysis of surfactin, which is sometimes reported as antibiotically active, did not show activity in our MIC tests, but showed synergistic effects combined with macrolactin A. The production of both compounds was clearly triggered by the presence of another strain, since in a bioactivity-guided isolation approach none of the compounds would have been caught from single cultures. The co-culture approach, based on the interaction of microbes can be regarded as an effective approach for the induction of secondary metabolites. Many studies in mixed microbe fermentation cultures have described triggered metabolite production. The co-culture of the marine-derived fungi *Emericella* sp. (strain CNL-878) and the marine actinomycete *Salinispora arenicola* (strain CNH-665) revealed markedly enhanced production of emericellamide A and emericellamide B^52^. A number of secondary metabolites were induced by co-culture of two sponge-derived Actinomycetes, *Actinokineospora* sp. EG49 and *Nocardiopsis* sp. RV163^53^. Yu et al*.*^54^ detected the chromone derivative 7-methoxy-2,3-dimethylchromone-4-one produced by *Streptomyces rochei* MB037. Co-culture with the gorgonian-derived fungus *Rhinocladiella similis* 35 stimulated its production significantly. It can be speculated that increased natural product production is caused by microbial competition for nutrients or space.

Surfactins are cyclic lipopeptides with surfactant-like properties, interfering with many kinds of cell membranes. They were reported to show antibacterial activity in agar diffusion assays against various test strains if used at high concentrations^55,56^. However, these results must be interpreted with caution, since the MICs determined were > 1024 µg/mL^56^, indicating no activity. The debate on the antimicrobial effect of surfactins remains active, but may be accounted to the non-specific mode of action on the cell layer, e.g. cation transport through the bilipid layer^57^, pore-formation on the cell wall^58^, and distortion of the membrane integrity by detergent-like properties of surfactins that induce membrane depolarization events in the host^59^. In consequence the cell’s energy balance is affected by compromising the cell wall potential, which results in reduced cell proliferation fitness and inactivation of transport processes into and out of the cell. Furthermore, deficient cell ultrastructure integrity may no longer block diffusion of large molecules, while its efflux efficiency may be severely reduced. A synergistic combination of sub-MIC surfactin and macrolactin A may be explained by this route. Synergistic effects of lipopeptides are described in some studies, *i.e.* surfactin with iturin , surfactin with fengycin, and iturin with fengycin^60^. In another study, synergistic effects between C15 surfactin and ketoconazole were described against *Candida albicans* SC5314^61^.

Macrolactins are macrolide compounds containing a 24-membered lactone ring, which are well known for their broad bioactivity spectrum like antiviral and anticancer properties, as well as activity against multi-resistant and clinically relevant pathogenic bacteria such as MRSA^62,63^. However, information about the common mode of action of this macrolide antibiotic remains scarce. A bacteriostatic mode of action against staphylococci and MRSA could be inferred from morphological changes during cell septum formation, which indicated cell wall synthesis inhibition as in the case of 7-*O*-malonyl macrolactin A^62^. As known for macrolide antibiotics, macrolactin N targets protein biosynthesis by inhibition of peptide deformylase in *S. aureus*^63^.

## Conclusions

The microbiome of marine sponges is a promising bioresource for natural products with various activities. An agar plate-based competition assay was performed for the selection of antibiotic-producing strains using inexpensive and easily accessible equipment and materials. Using BOX PCR in addition to 16S rRNA gene sequencing for the taxonomic identification, it was shown that diverse strains are selected by the competition assay. Integrating the analysis of the metabolic profile by comparing the cosine similarity of the strain extracts revealed that (i) strains selected from different sponges group together, which might be an indication that a specific metabolome is of ecological relevance. However, (ii) also different strains were selected by the competition assay, pointing towards different options in shaping a microbiome that can contribute to chemical defence. It was shown that molecules with assumed antibacterial effects, e.g. surfactins, are inactive alone (at the concentrations tested), but can have synergistic effects combined with other molecules produced by *Bacillus* species.

## Supplementary information

Supplementary information.
