# Supplementary material for: Selection of sponge-associated bacteria with high potential for the production of antibacterial compounds
Source: Sci Rep. 2020 Nov 12;10:19614. doi: 10.1038/s41598-020-76256-2 (PMC7665026; doi:10.1038/s41598-020-76256-2)
Supplement: Supplementary file 1 — Supplementary information. [file 41598_2020_76256_MOESM1_ESM.docx]

**Supplementary Information**

**Selection of sponge-associated bacteria with high potential for the production of antibacterial compounds**

*Riyanti^1,2^, Walter Balansa^3^, Yang Liu^1^, Abha Sharma^1^, Sanja Mihajlovic^4^, Christoph Hartwig^4^, Benedikt Leis^4^, Frets Jonas Rieuwpassa^3^, Frans Gruber Ijong^3^, Heike Wägele^5^, Gabriele M. König^6^, Till F. Schäberle^1,4,7*^*

^1^ Institute for Insect Biotechnology, Justus-Liebig-University of Giessen, 35392 Giessen, Germany;

^2^ Faculty of Fisheries and Marine Science, Jenderal Soedirman University, 53122 Purwokerto, Indonesia;

^3^ Department of Fisheries and Marine Science, Politeknik Negeri Nusa Utara 95821, North Sulawesi, Indonesia;

^4^ Fraunhofer Institute for Molecular Biology and Applied Ecology (IME), Branch for Bioresources, 35392 Giessen, Germany;

^5^ Centre of Molecular Biodiversity, Zoological Research Museum Alexander Koenig, 53113 Bonn, Germany;

^6^ Institute for Pharmaceutical Biology, University of Bonn, 53115 Bonn, Germany;

^7^ German Center for Infection Research (DZIF), Partner Site Giessen-Marburg-Langen, Giessen, Germany

**Content**

**Supplementary Methods**

- **Sponge collection and bacteria isolation**
- **Sponge spicule preparation and measurement**

**Supplementary Figures**

- **Figure S1.** LCHRMS of macrolactin A
- **Figure S2**. ^1^H NMR spectrum of macrolactin A
- **Figure S3**. HSQC spectrum of macrolactin A
- **Figure S4**. COSY correlations of macrolactin A
- **Figure S5**. HMBC spectrum of macrolactin A
- **Figure S6**. ^13^C NMR spectrum of macrolactin A
- **Figure S7**. LCHRMS of C14-surfactin and C15 surfactin
- **Figure S8**. ^1^H NMR spectrum of C14 surfactin
- **Figure S9**. ^1^H NMR spectrum of C15 surfactin
- **Figure S10**. LCHRMS of C16 surfactin and C17 surfactin
- **Figure S11.** Number of strains isolated from 10 sponges collected from Sangihe island, Indonesia based on media (a) and host sponges (b)
- **Figure S12.** Metabolome verification of one sample each metabolic group from 25 selected bacterial strains and medium control
- **Figure S13.** Cosine similarities of chemical fingerprints from 25 strains and medium control. All MS-runs performed are represented. The different samples are color-coded
- **Figure S14.**Molecular networking analysis of strain *Bacillus* sp. EP7-200 with the identification of C12, C14, C15 surfactin and lichenysin A
- **Figure S15.** Molecular networking analysis of strain *Bacillus* sp. EP5-815 with the identification of C12, C14, C15 surfactin and lichenysin A
- **Figure S16.** Molecular networking analysis of strain *Bacillus* sp. EP6-816 with the identification of C15 surfactin
- **Figure S17**. High performance liquid chromatography (HPLC) profiles of the EtOAc extract of different culture approaches pure culture of *Bacillus* sp. EP6-817 (A), pure culture of *L. sphaericus* EP6-121 (B) and co-culture *Bacillus* sp. EP6-817 and *L. sphaericus* EP6-121 (C). A clear increase in the production of one compound was observed during co culture (red line)
- **Figure S18.** Combination effect between macrolactin A and C14 surfactin against *Staphylococcus aureus* (ATCC 33592, MRSA). Data shows average values from 4 measurements ± 1x standard deviations

**Supplementary Tables**

- **Table S1.** GenBank accession numbers for the sequences reported in this paper
- **Table S2.** Morphological description of sponges and underwater documentation
- **Table S3.** Primary screening of antibacterial activity of 108 isolates against Gram-negative (*Escherichia coli*) and Gram-positive bacteria (*Micrococcus luteus*)
- **Table S4.** Competition assay between 25 high competitor strains originating from different sponges (diameter inhibition zone in mm red color)
- **Table S5.** Grouping output of the complete dataset 25 selected bacterial strains and the medium control
- **Table S6.** Inspected metabolic groups
- **Table S7.** Antibacterial activity 5 strongest strains against Gram negative bacteria (*E. coli*) and Gram-positive bacteria (*M. luteus*) in 10 different media
- **Table S8.** Data assessment microbroth dilution test of 5 compounds
- **Table S9.** Screening combination effect between macrolactin A and C14 surfactin

**Supplementary Methods**

**Sponge collection and bacteria isolation**

All specimens were photo-documented under water (GoPro Hero 4.0), transferred to the laboratory in Politeknik Nusa Utara (Polnustar), then cut and kept in a sterile plastic bag at -16°C. Small pieces were used for subsequent morphologically identification (Supplementary Methods and Table S1). All specimens (labelled EP1 to EP15, each ~500 g wet weight) were individually sliced into small pieces, dried in the oven at 45°C for 3 days and blended to give either powder or mash of sponges (on average ~30% of the wet weight).

To sponge material 500 µL of sterile distilled water was added; afterwards diluted 1:100 and plated on agar plates. Ten different agar media were used for isolation, the compositions per 1 L were: **(1) Marine Broth** (Carl Roth GmbH, Germany) with 12.5 µg/mL chloramphenicol added; **(2) Marine Broth** with 25 µg/mL nalidixic acid added; **(3) Marine Broth** with 25 µg/mL cycloheximide added; **(4) International Streptomyces Project (ISP) 2** with 2% NaCl: glucose (4g), malt extract (10g), yeast extract (4g); **(5) ISP2** with artificial sea water (ASW); **(6) M1** with ASW: starch (10g), yeast extract (4g), peptone (2g); **(7) Malt Yeast Extract (MYE)** with ASW: glucose (10g), malt extract (3g), yeast extract (3g), peptone (5g); **(8) Starch Nitrate (SN)** with ASW : starch (20g), KNO_3_ (1g), K_2_HPO_4_ (0.5g), MgSO_4_.7H_2_O (0.5g),NaCl (0.5g), FeSO_4_.7H_2_O (0.01g); **(9) Nutrient Agar** **(NA)**: peptone (5g), malt extract (3g), NaCl (5g); **(10)** **ASW** agar (20g) with *Escherichia coli* as prey bacteria. 25 µl of an *E. coli* overnight culture in LB medium was dropped onto ASW agar. Then, 10 µL of diluted sponge sample was dropped onto that spot on the agar. In order to minimize fungal growth, media 4-10 were supplemented with cycloheximide (25 µg/mL). The composition of 100% ASW per 1 L was: KBr (0.1g), NaCl (23.48g), MgCl_2_.6H_2_O (10.61g), CaCl_2_.2H_2_O (1.47g), KCl (0.66g), SrCl_2_.6H_2_O (0.04g), Na_2_SO_4_ (3.92g), NaHCO_3_ (0.19g), H_3_BO_3_ (0.03g). The plates were incubated at 30°C for 4 weeks. Colonies were selected based on different morphological characteristics and re-streaked on new plates until axenic cultures were obtained.

**Sponge spicule preparation and measurement**

To obtain spicules, a small piece (1 cm^3^) of each sponge was dried in the oven at 105°C for 1-2 h to completely remove water and subsequently left to cool down for 15 minutes. Maceration was performed in 5.0 mL commercial bleach (Bayclin, which 5% sodium hypochlorite) for 15 min. to 2 h, depending on toughness of specimens with the hard sponge normally requiring longer time. The sample was washed four times with distilled water and rinsed with alcohol (70%) to remove organic materials including sponge’s fibers. Following the maceration, the free spicules of each sponge were mounted on a microscope slide, air dried for few minutes and observed under a binocular microscope (Olympus, XSZ107BN) with 16x and 40x magnification for the ocular and objective lens respectively. All spicule’s pictures were taken with a Samsung J4+ 13-megapixel camerathrough the microscope ocular. The length and width of every spicule was determined by dividing its length and width—measured by the Corel Draw X6 (64-Bit) in cm on the spicule’s picture taken previously by the camera—with the total magnification value of the microscope (640 total magnification resulting from a 4x magnification of ocular and 60x of objective lens) and then multiplying the value by 10,000 to get the final value of the measurement in micrometre (μm) as indicated in the following spicule’s pictures at Supplementary Table S2.

**Supplementary Figures**

**Figure S1.** LCHRMS of macrolactin A


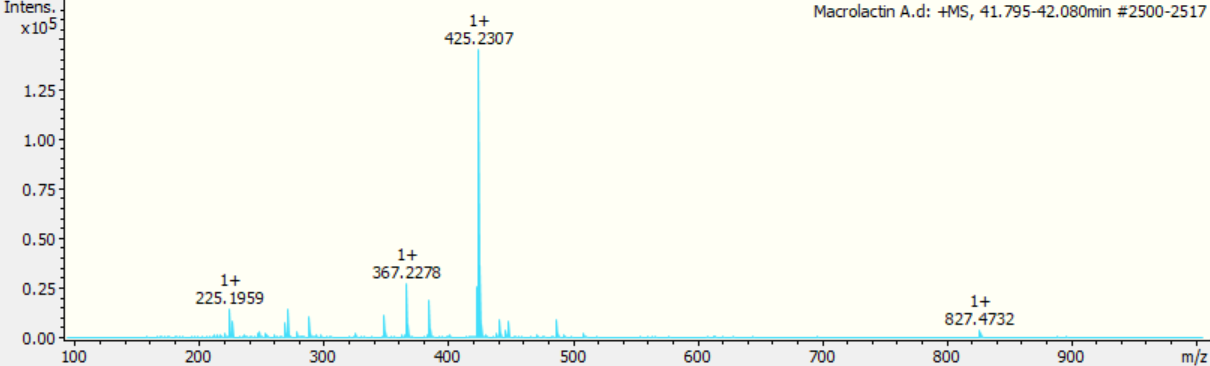


**Figure S2**. ^1^H NMR spectrum of macrolactin A
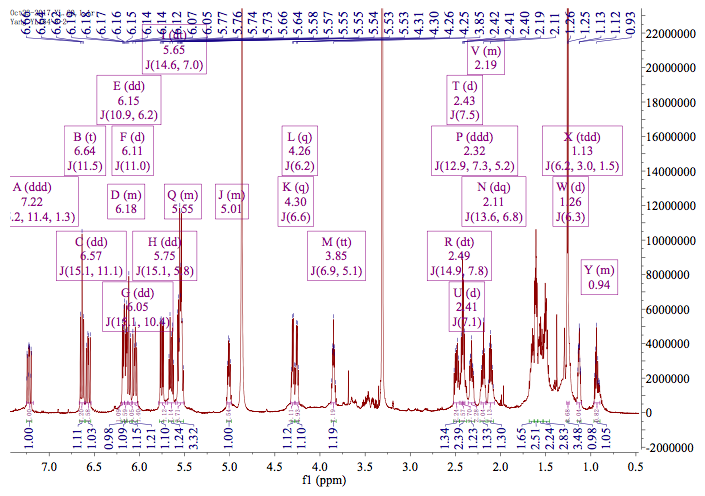


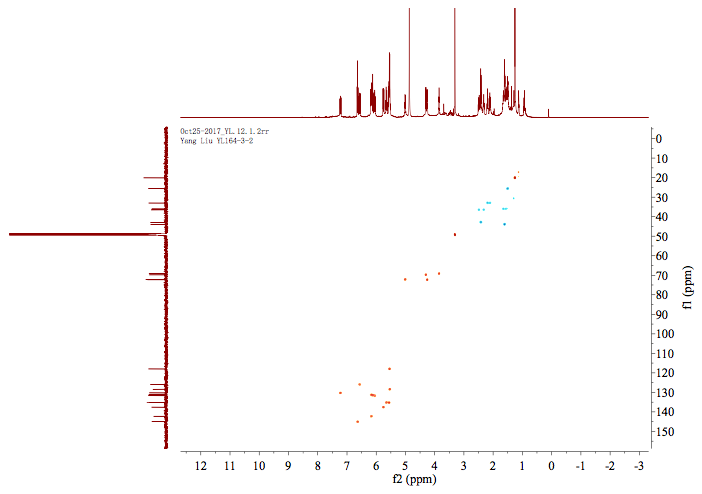
**Figure S3**. HSQC spectrum of macrolactin A

**Figure S4**. COSY correlations of macrolactin A
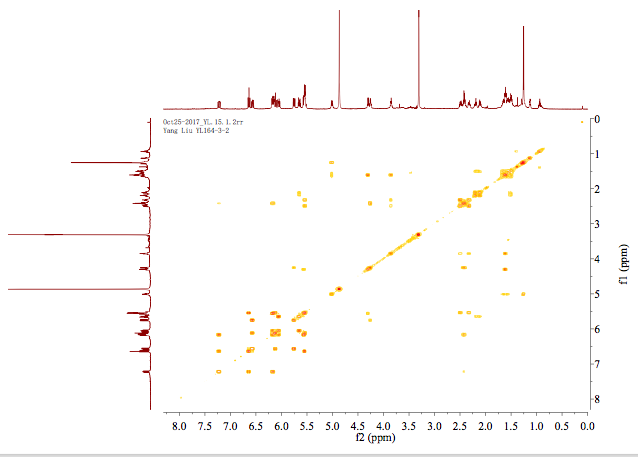


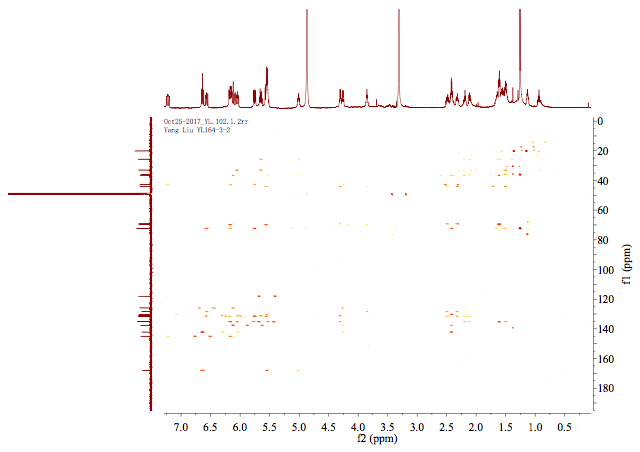
**Figure S5**. HMBC spectrum of macrolactin A


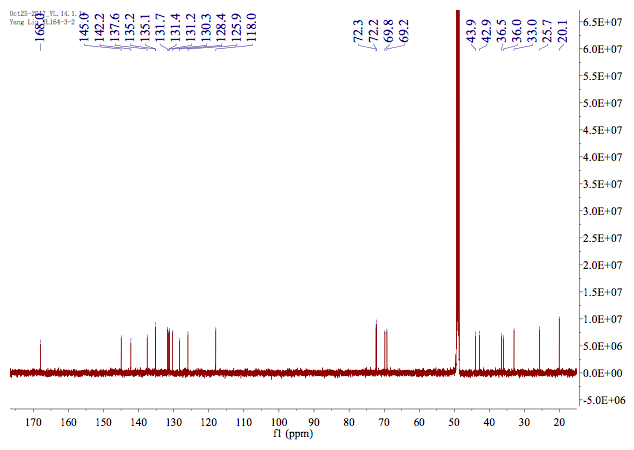
**Figure S6**. ^13^C NMR spectrum of macrolactin A

**Figure S7**. LCHRMS of C14-surfactin and C15 surfactin


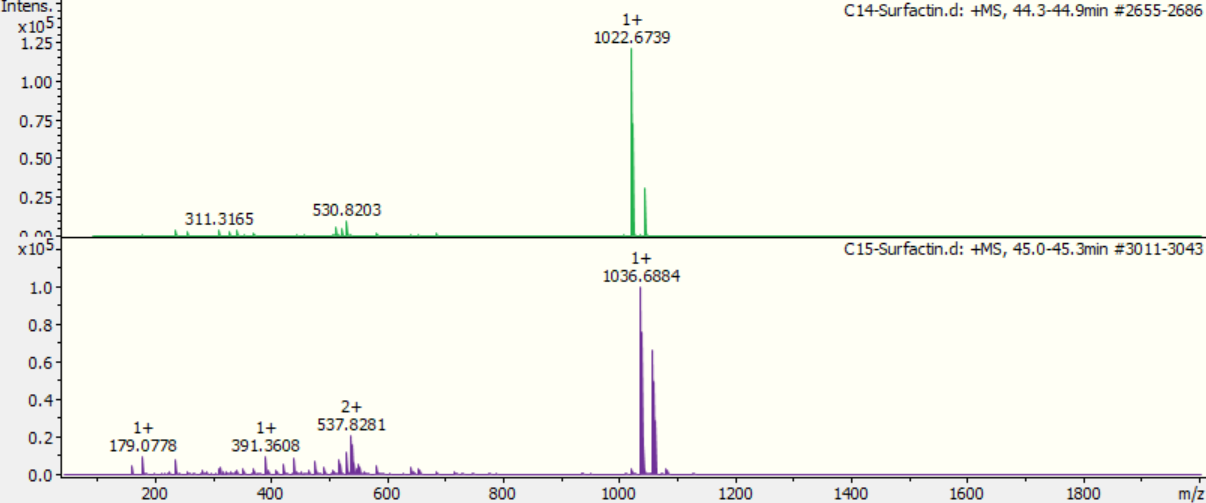


**Figure S8**. ^1^H NMR spectrum of C14 surfactin


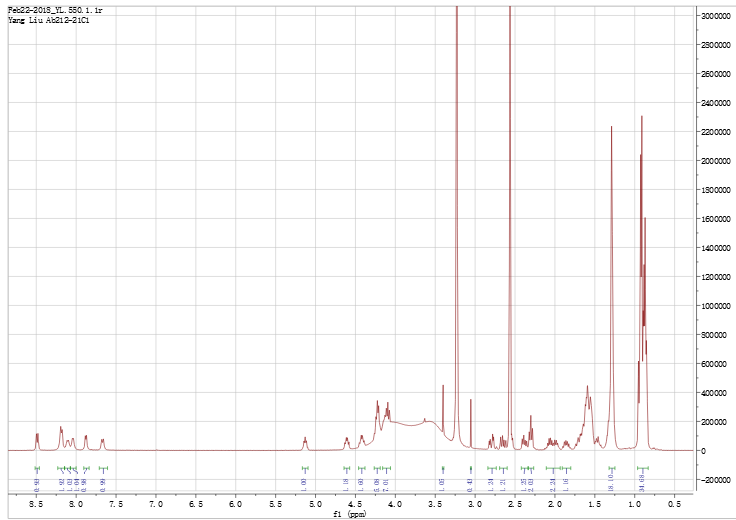


**Figure S9**. ^1^H NMR spectrum of C15 surfactin


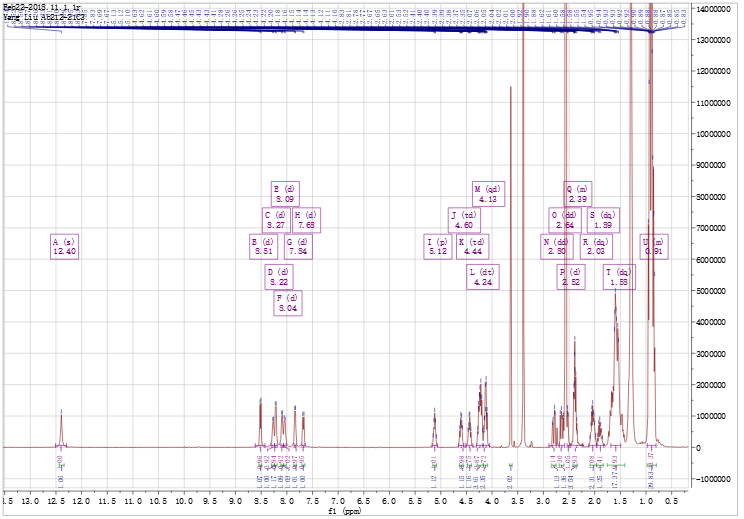


**Figure S10**. LCHRMS of C16 surfactin and C17 surfactin


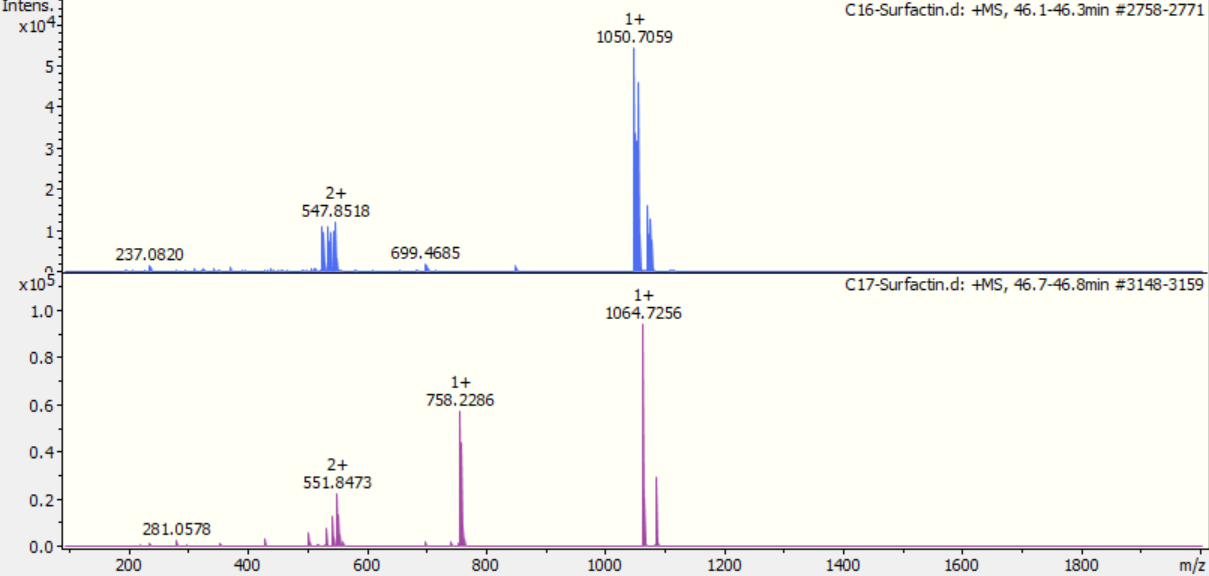


**Figure S11.** Number of strains isolated from 10 sponges collected from Sangihe Island, Indonesia based on media (a) and host sponges (b).

a

b

**Figure S12.** Metabolome verification of one sample each metabolic group from 25 selected bacterial strains and medium control.

Group 17 is represented by the first fermentation of *Bacillus* sp. EP6-195, which is deemed to be a co-culture and excluded in Figure 5.

**Figure S13.** Cosine similarities of chemical fingerprints from 25 strains and medium control. All MS-runs performed are represented. Strain-IDs and clades (according to box-plot in figure 4) are color-coded in the sidebars. Clustering is based on cosine similarity profiles.


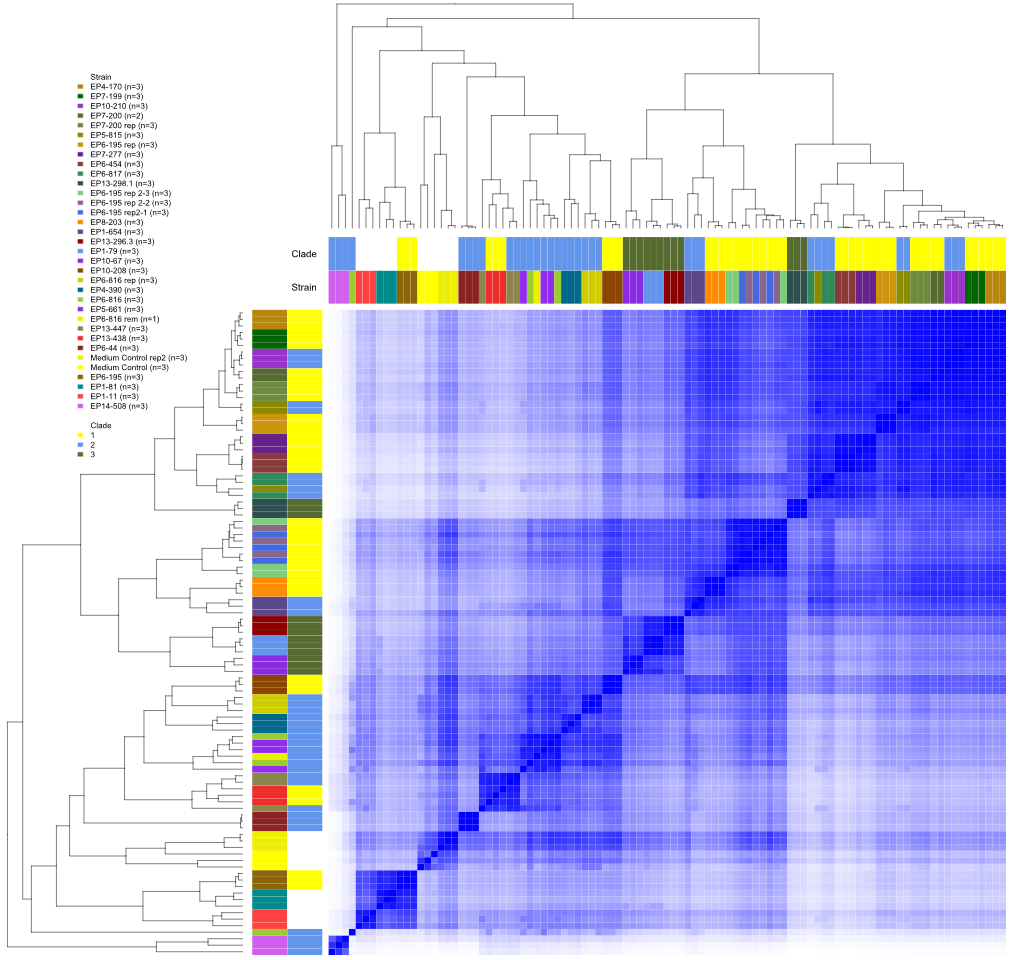


In Figure S13 the complete dataset is shown. One sample was remeasured (strain 186 rem) after storage and a couple of lipophilic peaks vanished, likely due to precipitation. It subsequently clustered in a different group. In first fermentation of EP7-200 one extract was lost, the refermentation was highly similar and is used for main analysis. EP6-816 was repeated to corroborate the clustering behaviour, the second round resulted in higher compound concentrations (and therefore clustered differently) but showed very similar pattern to first fermentation in visual MS-Data inspection. The second fermentation of EP6-816 is used for main analysis.

Strain EP6-195 was fermented in three batches. In the first fermentation it was highly similar to strain EP1-81. In the second round it clustered with the complete metabolic group 1, making contamination likely. For the third fermentation three cryo-stored starter cultures were used to ferment triplicates each, no other strain was handled in the vicinity during that time. All three triplicates are highly similar to each other, making the result trustworthy. The strain has a metabolomics profile similar to strains EP8-203 and EP1-654. However, these were not handled during third fermentation of EP6-195.

These results show that the strongest competitor approach needs careful handling to conserve diversity, and that a combination of genetic and metabolomic analysis helps in analysis of the results.

**Figure S14.** Molecular networking analysis of strain *Bacillus* sp. EP7-200

A


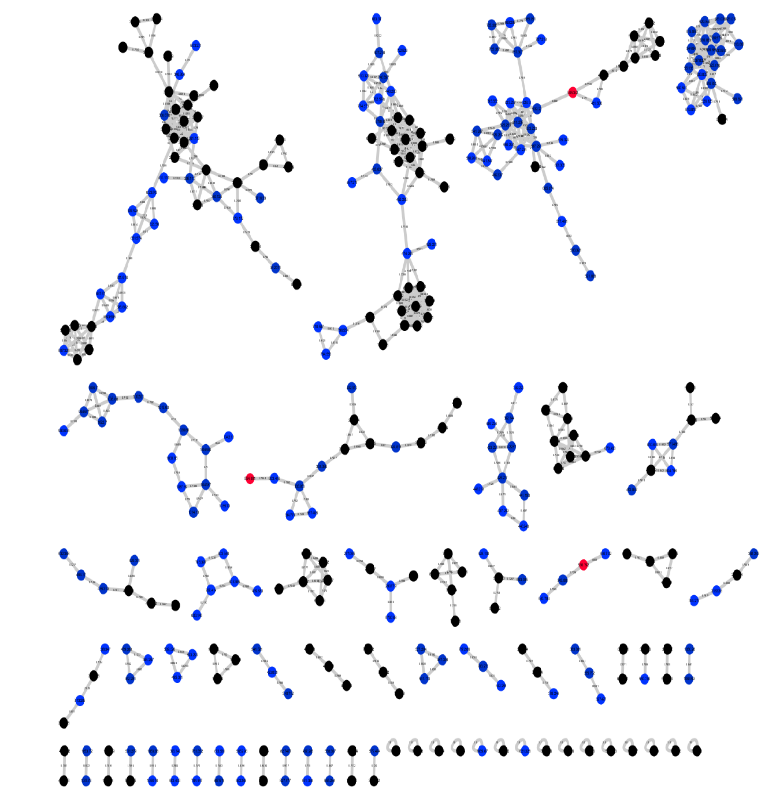

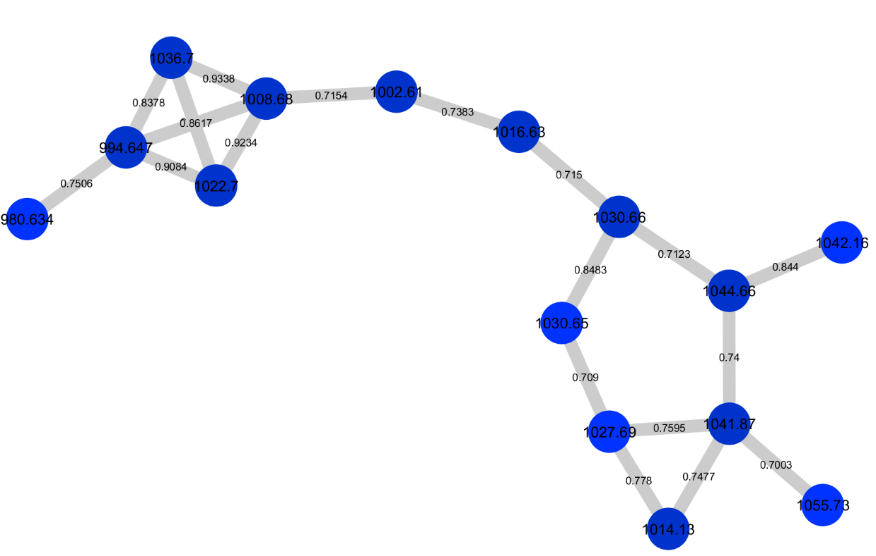


Lichenysin A

Surfactin C-15

Surfactin C-14

Surfactin C-12

BB

1. Colours indicate media and conditions when the respective ion was detected: Media background (black), present in all media (blue) and solely in ISP2 medium (red), of which the extract showed activity against *E. coli*. (B) Enlarged cluster of surfactins and lichenysin A, which were dereplicated.

**Figure S15.** Molecular networking analysis of strain *Bacillus* sp. EP5-815.

A


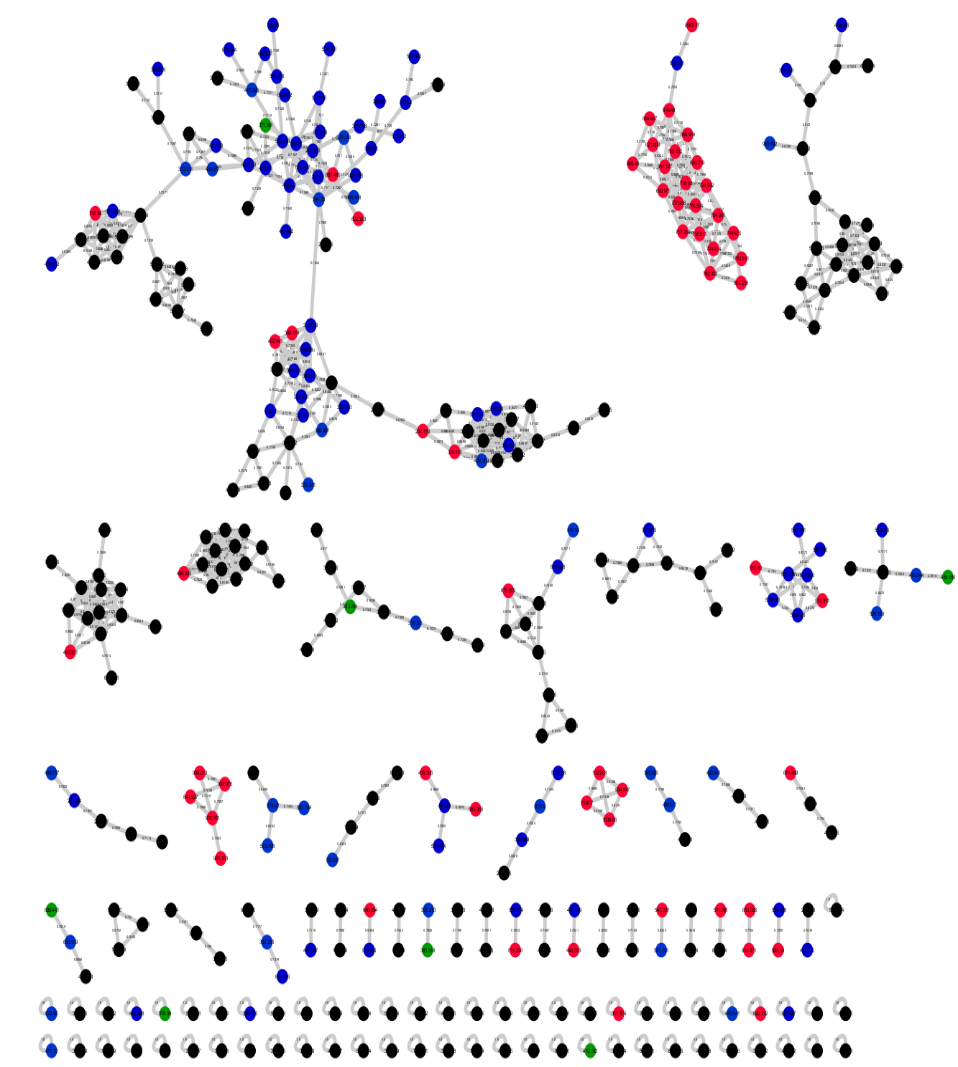

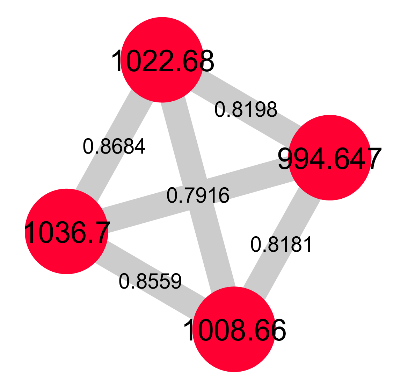


Surfactin C-14

Surfactin C-12

Lichenysin A

Surfactin C-15

B

1. Colours indicate the media and conditions when the respective ion was detected. Media (black), present in all media (blue), solely present in LB and NB media (green), of which the extract showed activity against *E. coli*, and MB medium (red). (B) Enlarged cluster of surfactins and lichenysin A, which were produced by *Bacillus* sp. EP5-815 in MB medium only.

**Figure S16.** Molecular networking analysis of strain *Bacillus* sp. EP6-816.

A

A


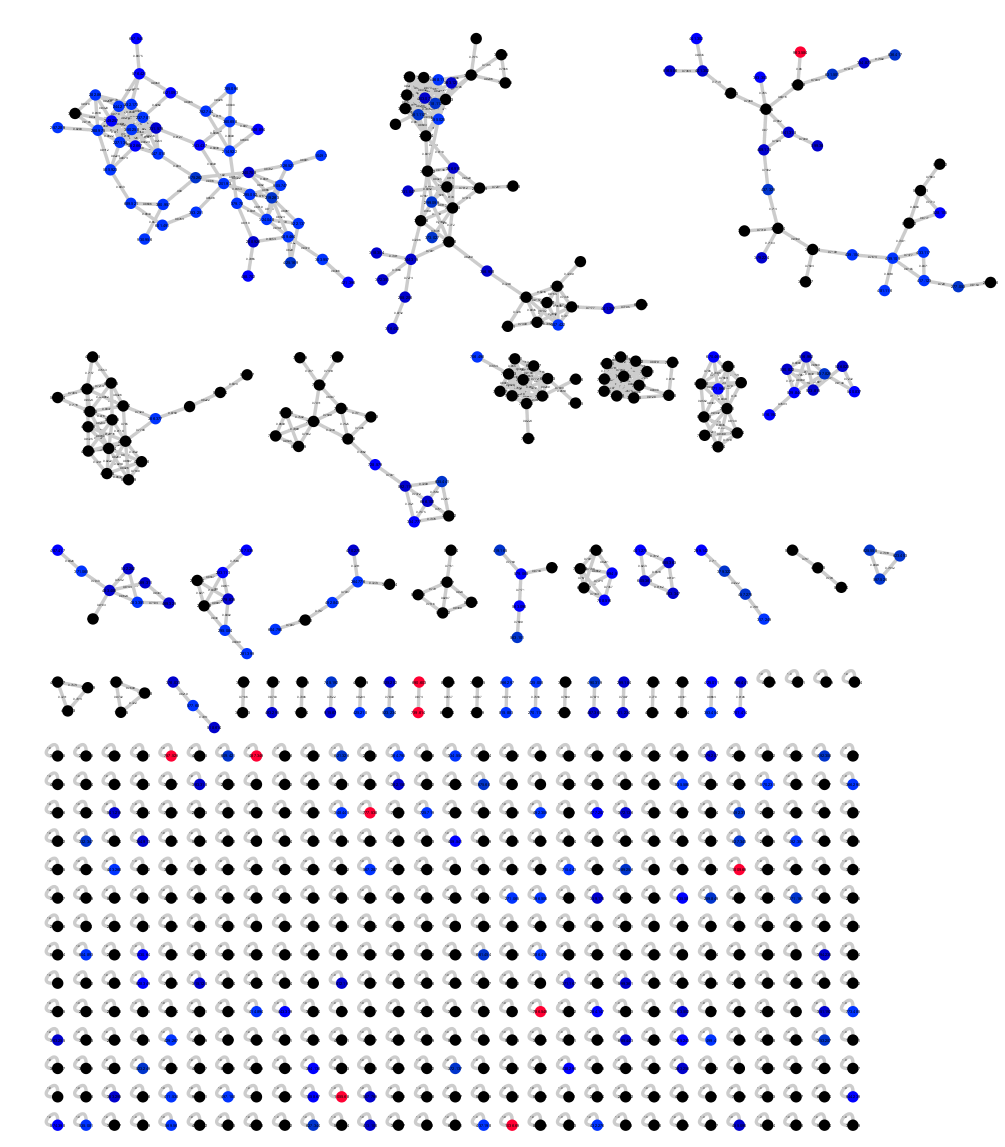


B


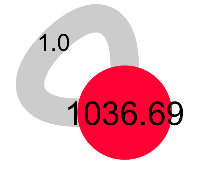


Surfactin C-15

1. Colours indicate the media and conditions when the respective ion was detected. Media (black), present in all media (blue), tryptone and yeast extract medium with ASW (red). (B) Enlarged cluster of C15 surfactin.

**Figure S17**. High performance liquid chromatography (HPLC) profiles of the EtOAc extract of different culture approaches pure culture of *Bacillus* sp. EP6-817 (A), pure culture of *L. sphaericus* EP6-121 (B) and co-culture *Bacillus* sp. EP6-817 and *L. sphaericus* EP6-121 (C). A clear increase in the production of one compound was observed during co culture (red line).


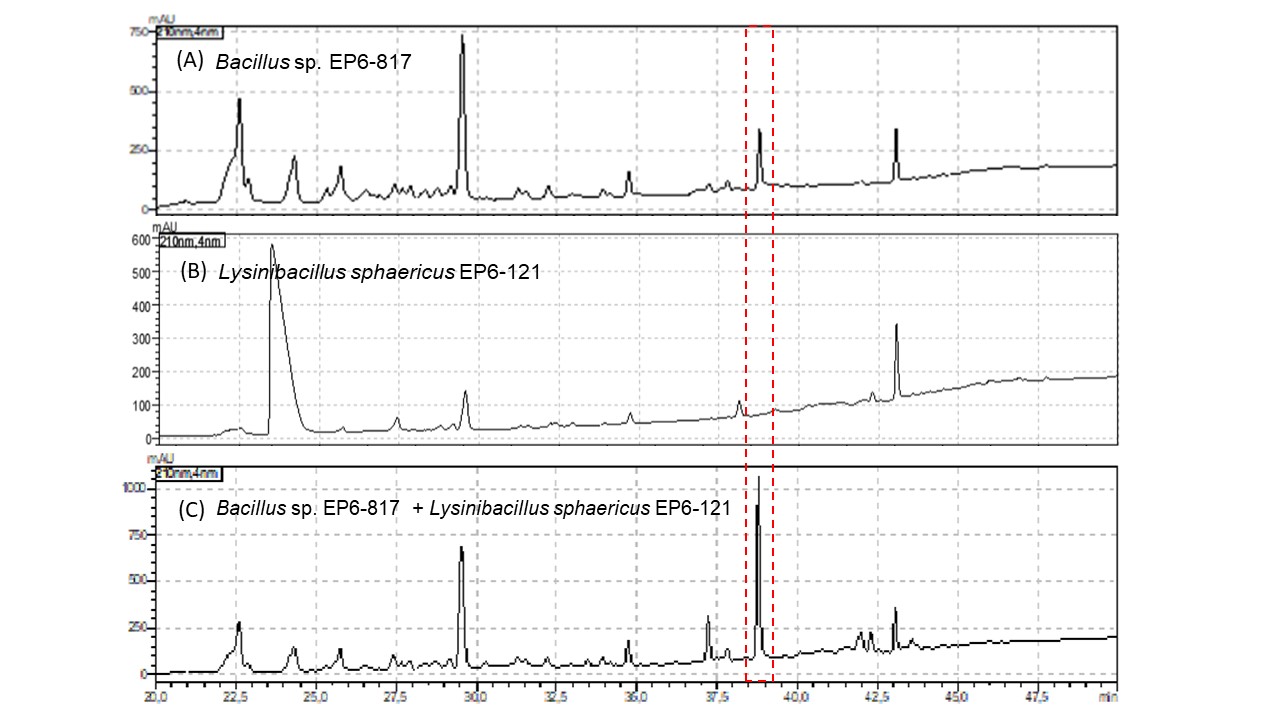


**Figure S18**. Combination effect between macrolactin A and C14 surfactin against *Staphylococcus aureus* (ATCC 33592, MRSA). Data shows average values from 4 measurements ± 1x standard deviations.

**Supplementary Tables**

**Table S1.** GenBank accession numbers for the sequences reported in this paper

| Strain | Accession Number |
| --- | --- |
| *Bacillus* sp. EP1-11 | MT314037 |
| *Bacillus thuringiensis* EP6-44 | MT314038 |
| *Bacillus tequilensis* EP5-661 | MT314039 |
| *Bacillus stratosphericus* EP1-79 | MT314040 |
| *Bacillus* sp. EP7-277 | MT314041 |
| *Bacillus* sp. EP8-203 | MT314042 |
| *Bacillus* sp. EP10-208 | MT314043 |
| *Bacillus* sp. EP6-195 | MT314044 |
| *Bacillus subtilis* EP7-199 | MT314045 |
| *Bacillus subtilis* EP7-200 | MT314046 |
| *Bacillus* sp. EP13-438 | MT314047 |
| *Bacillus* sp. EP1-654 | MT314048 |
| *Bacillus subtilis* EP4-170 | MT314049 |
| *Bacillus* sp. EP13-296.3 | MT314050 |
| *Bacillus sonorensis* EP13-298.1 | MT314051 |
| *Leclercia* sp. EP4-390 | MT314052 |
| *Brevibacterium* sp. EP14-508 | MT314053 |
| *Pseudomonas balearica* EP13-447 | MT314054 |
| *Bacillus* sp. EP10-67 | MT314055 |
| *Bacillus* sp. EP6-454 | MT314056 |
| *Bacillus* sp. EP5-815 | MT314057 |
| *Bacillus* sp. EP6-816 | MT314058 |
| *Bacillus* sp. EP6-817 | MT314059 |
| *Verrucosispora* sp. EP6-325 | MT314060 |
| *Lysinibacillus sphaericus* EP6-121 | MT314061 |

**Table S2.** Morphological description of sponges and underwater documentation

| **Code** | **Morphological features** | **Image** | **Type of Spicule** | **Ref** |
| --- | --- | --- | --- | --- |
| **EP1** | Sponge orange coloured underwater (a) and also after exposure to air (b); soft, with a foliaceous (leaf like shaped) growth form and corrugated surface structure. Morphological appearance and spicule type (c) suggest assignment to *Stylissa flabelliformis*. Collected in shallow water (~1 m) of a coral reef from a rocky bottom. | 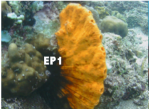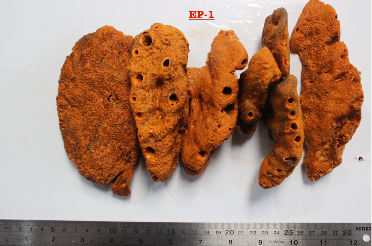 *a*  *b*  *Stylissa flabelliformis* | 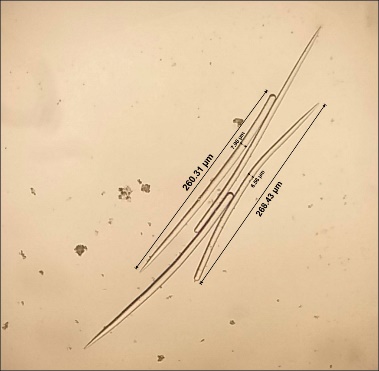*c*  Megascleres, type Style (with one end pointed and the other end round) (277.65 - 286.33 µm) | ^1^  ^2^  ^3^ |
| **EP4** | Sponge dark orange coloured underwater and encrusting (a), very small conulate or cone-shaped projections. Morphological appearance and spicule type (b) suggest assignment to *Petrosia* sp.  Collected at ~4 m, spreading over rocky bottom. | 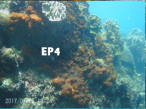*a*  *Petrosia* sp. | 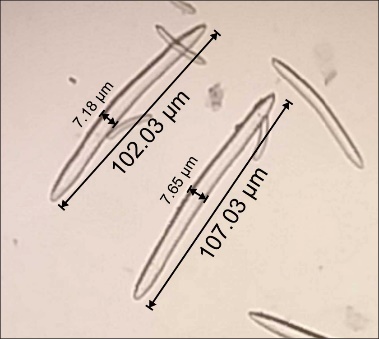*b*  Megascleres, type Oxea with both ends pointed (102.03 x 7.18 − 107.03 x 7.65); Microscleres of Oxea type are also present. |  |
| **EP5** | Sponge large and barrel-shaped, with ribbed surface and a deep cavity in the centre; incompressible (hard) (a). Morphological appearance and spicule type (b) suggest assignment to *Xestospongia testudinaria.* Collected at ~7 m. | *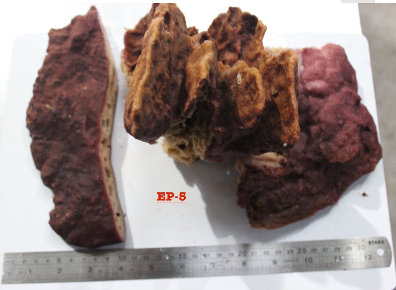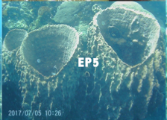a*  *b*  *Xestospongia testudinaria* | 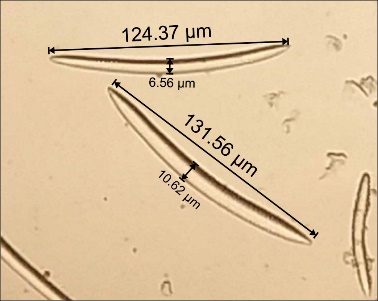*b*  Megascleres, type Oxea (124.37 x 6.56−131.56 x 10.62 µm) | ^4^ |
| **EP6** | Sponge black coloured underwater, shape amorphous (a). Morphological appearance and spicule type (b) suggest assignment to *Neopetrosia sp*. Collected at ~5 m. | *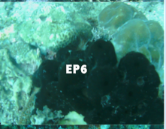a*  *Neopetrosia sp* | 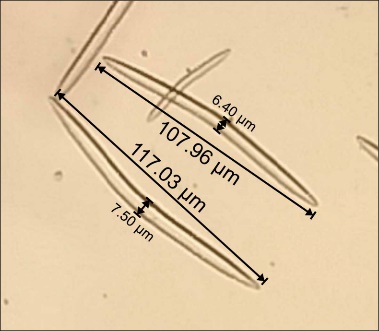*b*  Megascleres, type Oxea (107.96 x 6.40-117.03 − 117.03 x 7.50 µm); microscleres of Oxea type also present |  |
| **EP7** | Large sponge of grey color underwater (a), dark grey (b) and light brown (c) when exposed to air; body shape spherical with a spiny fibrous exterior surface structure, but soft interior; very tough, like rubber, and very smelly. Morphological appearance and lack of any spicules suggest assignment to *Ircinia strobilina.* Collected at ~6 m on a rocky bottom. | 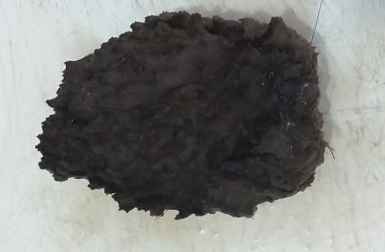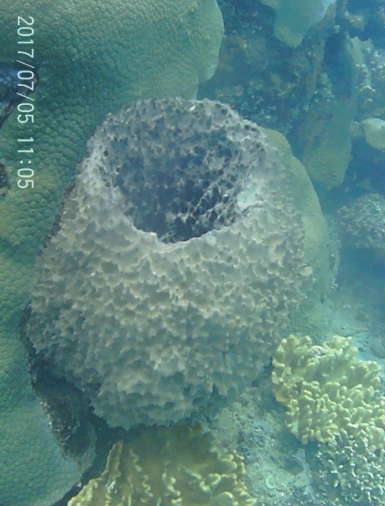*a*  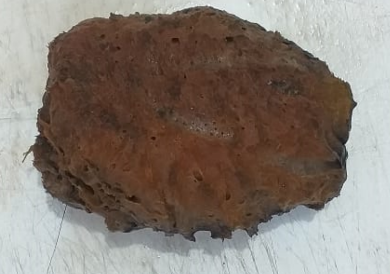*b*  *c*  *Ircinia strobilina* | Mineral spicules absent. | ^5^  ^6^  ^7^ |
| **EP8** | Sponge black; shape encrusting, lobate and fragile; (a), producing slime, which is typically released as a black and purple exudate when damaged (b). Morphological appearance and spicule type (not figured) suggest assignment to *Neopetrosia* sp. Collected at ~4 m. | *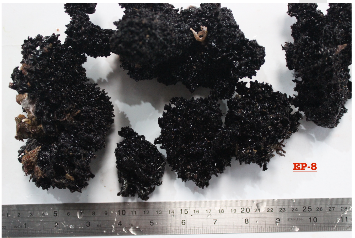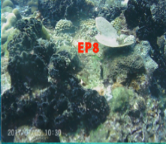a*  *b*  *Neopetrosia* sp. | Three types of Megascleres, type Strongyles (thin, with both ends blunt or round), type Style, and a very thin type (107.03 x 5.46-107.3 x 5.62 µm). | ^8^  ^9^  ^10^ |
| **EP10** | Sponge reddish to black exteriorly and bright yellow inside; massive, with bluntly lobate form (a). Morphological appearance and spicule type (b) suggest assignment to *Aaptos suberitoides*. Collected at ~5 m. | 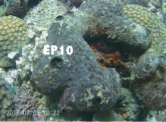*a*  *Aaptos suberitoides* | 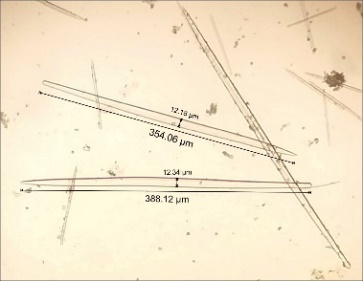*b*  Megascleres, type Strongyloxea (379.83-416.66 µm) | ^11^  ^12^ |
| **EP13** | Sponge greenish brown, soft, with massive oscular chimneys (fistules); surface structure uneven and brittle (a). Collected at ~5 m on coral sandy bottom. Morphological appearance and spicule type suggest assignment to *Spechiosponiga vagabunda*. *(Remark: Morph. appearance would also fit to* A. ingens; *however,*  *subtylostyle megascleres are not described for the latter.)* | *a* 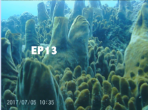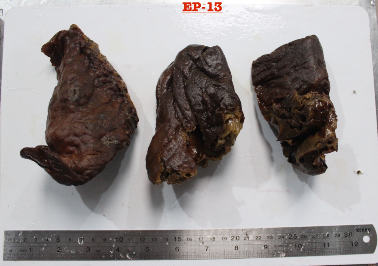 *Spechiosponiga vagabund* | 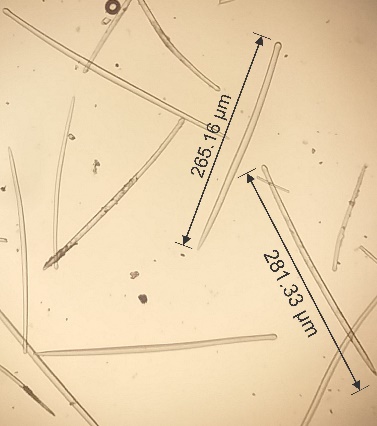b  Megascleres, type Subtylostyle (265.16 -281.33 µm) | ^13^  ^14^ |
| **EP14** | Sponge light brown underwater, redish brown when exposed to air (a); egg-shaped with a large osculum in the middle. Morphological appearance and spicule type (b) suggest assignment to *Melophus sarasinorum*. Collected at a depth of ~5 meter. | *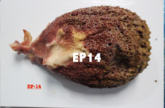a*  *Melophlus/Asteropus sarasinorum* | 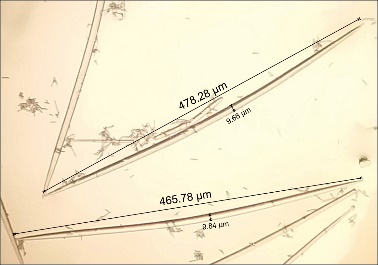b  Megascleres, type Oxea, very thin (498.22-515.16 µm) | ^15^ |
| **EP15** | Sponge reddish-brown to brick red, with the interior of similar, but lighter colour, especially when dry; massive and round in shape, with encrusting growth form; hardly compressible (a). Based on DNA barcoding analysis Balansa *et al*. (2020) identified this specimen as *Agelas* sp. Morphological appearance and spicule type (b) suggest assignment to *Agelas nakamurai*. | 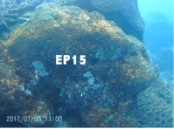a  *Agelas nakamurai* | 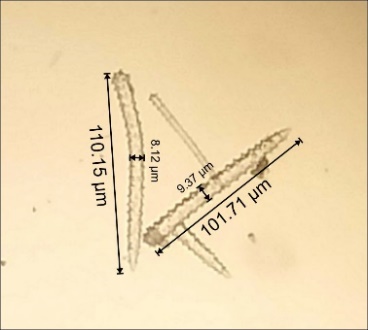b  Megascleres, type  Acanthostyle (101.71 x 9.37-110.15 x 8.12 µm) | ^16^ |

**Table S3.** Primary screening of antibacterial activity of 108 isolates against Gram-negative (*Eschericia coli*) and Gram-positive bacteria (*Micrococcus luteus*)

| **No** | **Isolates** | **Activity** | |
| --- | --- | --- | --- |
|  |  | ***E. coli*** | ***M. luteus*** |
| 1 | *Bacillus* sp. EP1-11 | - | +++ |
| 2 | *Bacillus cereus* EP4-29 | - | +++ |
| 3 | *Pseudomonas balearica* EP4-31 | - | +++ |
| 4 | *Bacillus* sp. EP6-35 | - | + |
| 5 | *Pseudomonas* sp. EP6-36 | - | ++ |
| 6 | *Bacillus thuringiensis* EP6-44 | - | + |
| 7 | *Bacillus aerius* EP10-66 | - | ++ |
| 8 | *Bacillus* sp. EP10-67 | ++ | - |
| 9 | *Bacillus* sp. EP13-69 | - | ++ |
| 10 | *Bacillus stratosphericus* EP1-79 | - | ++ |
| 11 | *Lysinibacillus* sp. EP1-81 | - | ++ |
| 12 | *Lysinibacillus sphaericus* EP1-82 | - | ++ |
| 13 | *Bacillus altitudinis* EP5-102 | - | + |
| 14 | *Serratia* sp. EP5-103.1 | - | + |
| 15 | *Bacillus subtilis* EP5-103.2 | - | + |
| 16 | *Lysinibacillus sphaericus* EP5-105 | - | +++ |
| 17 | *Bacillus indicus* EP4-109 | - | + |
| 18 | *Bacillus* sp. EP4-112 | - | ++ |
| 19 | *Pseudomonas* sp.  EP4-113 | - | +++ |
| 20 | *Staphylococcus* sp. EP6-114 | - | + |
| 21 | *Bacillus safensis* EP6-119 | - | + |
| 22 | *Bacillus altitudinis* EP6-120 | - | + |
| 23 | *Lysinibacillus sphaericus* EP6-121 | - | + |
| 24 | *Bacillus stratosphericus* EP6-122 | - | + |
| 25 | *Bacillus aerius* EP6-123 | - | + |
| 26 | *Bacillus aerius* EP6-124 | - | + |
| 27 | *Staphylococcus* sp. EP6-125 | - | + |
| 28 | *Bacillus cereus* EP7-128 | - | ++ |
| 29 | *Bacillus cereus* EP7-133 | - | +++ |
| 30 | *Bacillus aerophilus* EP10-141 | - | + |
| 31 | *Pseudomonas* sp. EP10-142 | - | + |
| 32 | *Bacillus altitudinis* EP13-159 | - | ++ |
| 33 | *Bacillus* sp. EP1-163 | - | +++ |
| 34 | *Bacillus* sp. EP1-164 | - | +++ |
| 35 | *Bacillus subtilis* EP4-170 | - | + |
| 36 | *Bacillus* sp. EP5-180 | - | ++ |
| 37 | *Bacillus* sp. EP5-181 | - | ++ |
| 38 | *Bacillus* sp. EP6-195 | + | +++ |
| 39 | *Bacillus subtilis* EP7-199 | +++ | +++ |
| 40 | *Bacillus subtilis* EP7-200 | +++ | +++ |
| 41 | *Bacillus subtilis* EP7-202 | - | ++ |
| 42 | *Bacillus* sp. EP8-203 | - | +++ |
| 43 | *Bacillus* sp. EP10-208 | +++ | +++ |
| 44 | *Lysinibacillus fusiformis* EP10-209 | - | + |
| 45 | *Bacillus subtilis* EP10-210 | - | + |
| 46 | *Enterobacter* sp. EP4-251 | - | + |
| 47 | *Enterobacter* sp. EP4-255 | - | + |
| 48 | *Bacillus zhangzhouensis* EP5-268 | - | + |
| 49 | *Staphylococcus kloosii* EP5-270 | - | + |
| 50 | *Bacillus amyloliquefaciens* EP7-273 | - | + |
| 51 | *Bacillus* sp. EP7-274 | - | + |
| 52 | *Bacillus* sp. EP7-277 | - | + |
| 53 | *Bacillus velezensis* EP7-278 | - | ++ |
| 54 | *Staphylococcus* sp. EP10-289 | - | + |
| 55 | *Staphylococcus* sp. EP14-293 | - | + |
| 56 | *Cronobacter sakazakii* EP13-296.3 | - | + |
| 57 | *Bacillus sonorensis* EP13-298.1 | - | ++ |
| 58 | *Bacillus* sp. EP4-304 | - | +++ |
| 59 | *Bacillus* sp. EP4-309 | - | + |
| 60 | *Enterobacter* sp. EP4-311 | - | + |
| 61 | *Bacillus* sp. EP5-314 | - | + |
| 62 | *Serratia* sp. EP5-317 | - | ++ |
| 63 | *Staphylococcus* sp. EP6-323 | + | - |
| 64 | *Bacillus* sp. EP6-324 | + | - |
| 65 | *Verrucosispora* sp. EP6-325 | + | - |
| 66 | *Bacillus cereus* EP7-334 | - | +++ |
| 67 | *Bacillus* sp. EP13-345 | - | +++ |
| 68 | *Bacillus* stratosphericus EP13-347 | - | +++ |
| 69 | *Citrobacter sedlakii* EP4-372 | - | + |
| 70 | *Bacillus* sp. EP1-379 | + | + |
| 71 | *Serratia* sp. EP1-388 | + | + |
| 72 | *Leclercia* sp. EP4-390 | - | ++ |
| 73 | *Bacillus* sp. EP4-391 | + | +++ |
| 74 | *Bacillus altitudinis* EP6-405 | - | + |
| 75 | *Bacillus tequilensis* EP6-407 | - | + |
| 76 | *Citrobacter sedlakii* EP6-409 | - | + |
| 77 | *Bacillus altitudinis* EP6-410 | - | + |
| 78 | *Bacillus velezensis* EP7-417 | ++ | ++ |
| 79 | *Bacillus aerius* EP7-419 | ++ | - |
| 80 | *Bacillus amyloliquefaciens* EP7-424 | + | ++ |
| 81 | *Bacillus* sp. EP7-428 | - | + |
| 82 | *Bacillus* sp. EP13-438 | - | +++ |
| 83 | *Pseudomonas balearica* EP13-447 | - | ++ |
| 84 | *Citrobacter sedlakii* EP13-449 | - | + |
| 85 | *Bacillus* sp. EP6-454 | + | ++ |
| 86 | *Brevibacterium* sp. EP14-508 | - | + |
| 87 | *Bacillus subtilis* EP1-652 | - | ++ |
| 88 | *Bacillus* sp. EP1-653 | - | ++ |
| 89 | *Bacillus* sp. EP1-654 | - | +++ |
| 90 | *Bacillus velezensis* EP1-655 | + | +++ |
| 91 | *Bacillus velezensis* EP1-656 | + | +++ |
| 92 | *Bacillus velezensis* EP1-655.1 | + | +++ |
| 93 | *Bacillus velezensis* EP1-657 | + | +++ |
| 94 | *Bacillus* sp. EP1-658 | - | ++ |
| 95 | *Bacillus tequilensis* EP5-661 | - | ++ |
| 96 | *Bacillus velezensis* EP7-663 | - | +++ |
| 97 | *Bacillus* sp. EP7-664 | - | ++ |
| 98 | *Pseudomonas balearica* EP8-741 | - | + |
| 99 | *Pseudomonas* sp. EP8-742 | - | + |
| 100 | *Bacillus* sp. EP5-815 | - | ++ |
| 101 | *Bacillus* sp. EP6-816 | - | +++ |
| 102 | *Bacillus* sp. EP6-817 | - | +++ |
| 103 | *Solwaraspora* sp. EP8-524 | ++ | +++ |
| 104 | *Solwaraspora* sp. EP8-525 | ++ | ++ |
| 105 | *Solwaraspora* sp. EP8-532 | ++ | ++ |
| 106 | *Solwaraspora* sp. EP8-533 | ++ | ++ |
| 107 | *Solwaraspora* sp. EP8-540 | - | ++ |
| 108 | *Cellulosimicrobium* sp. EP1-15 | - | ++ |

- : no activity + : weak activity

++ : moderate activity +++ : strong activity

**Table S4.** Competition assay between 25 high competitor strains originating from different sponges (diameter inhibition zone in mm red colour)

| **Sponge** | **Sponge** | **EP 1** | | | | **EP 4** | | **EP 5** | | **EP 6** | | | | | **EP 7** | | | **EP 8** | **EP 10** | | | **EP 13** | | | | **EP 14** |
| --- | --- | --- | --- | --- | --- | --- | --- | --- | --- | --- | --- | --- | --- | --- | --- | --- | --- | --- | --- | --- | --- | --- | --- | --- | --- | --- |
|  | **Strain^a^** | **11** | **79** | **81** | **654** | **170** | **390** | **661** | **815** | **44** | **195** | **454** | **816** | **817** | **199** | **200** | **277** | **203** | **67** | **208** | **210** | **296** | **298** | **438** | **447** | **508** |
| **EP 1** | **11** | **x** |  |  | **10,0** |  |  |  |  | 11,0 |  | 10,0 |  |  |  |  |  |  |  |  |  |  |  |  |  |  |
|  | **79** |  | **X** | **10,0** | **9,0** |  |  |  |  |  |  | 13,0 |  |  |  |  |  |  |  |  |  |  |  |  |  |  |
|  | **81** |  |  | **x** |  |  |  |  | 10,0 | 11,0 |  |  | 11,0 |  |  |  |  |  | 11,0 | 12,0 |  |  |  |  |  | 12,0 |
|  | **654** | **13,3** | **13,3** | **20,0** | **x** | 13,0 |  | 12,0 |  | 12,0 | 20,0 | 9,0 |  | 10,0 |  |  | 9,0 |  | 20,0 |  | 9,0 | 14,3 | 10,0 | 12,0 |  | 22,0 |
| **EP 4** | **170** |  |  |  | **17,0** | x |  |  | 10,7 | 14,0 | 11,0 |  |  |  |  |  |  |  | 16,0 |  |  | 10,0 |  |  |  | 17,0 |
|  | **390** |  |  |  |  |  | X |  |  |  |  |  |  |  |  |  |  |  |  |  |  |  |  |  |  |  |
| **EP 5** | 661 |  |  |  |  |  |  | x |  |  |  |  |  |  |  |  |  |  |  |  |  |  |  |  |  |  |
|  | 815 | 9,3 | 10,7 | 9,0 | 18,0 | 9,0 |  |  | x | 9,0 | 15,0 |  |  |  |  |  |  |  | 11,3 |  |  | 12,7 |  | 15,3 | 15,3 | 19,0 |
| **EP 6** | 44 |  |  |  |  |  |  |  |  | x |  |  |  |  |  | 9,0 |  |  |  |  | 9,0 | 9,0 |  |  |  |  |
|  | 195 |  |  |  |  |  |  |  | 12,0 |  | x |  | 8,0 | 8,0 | 9,0 |  | 10,0 |  |  | 12,0 |  |  |  |  |  | 10,0 |
|  | 454 |  |  |  |  |  |  |  |  |  | x | 14,0 |  |  |  |  |  |  |  |  |  | 9,0 |  |  |  | 17,0 |
|  | 816 | 9,0 | 9,0 | 9,0 | 17,0 | 9,0 |  | 9,0 |  |  | 17,0 |  | x |  |  |  |  |  | 17,0 |  |  | 11,0 |  | 16,7 | 14,7 | 19,0 |
|  | 817 | 9,0 |  | 10,0 |  | 9,0 |  |  |  |  | 17,0 |  |  | x |  |  |  |  | 15,0 |  |  | 11,0 |  | 10,0 | 14,0 | 18,3 |
| **EP 7** | 199 | 9,0 | 9,0 | 9,0 | 17,3 | 9,0 |  | 10,0 |  |  | 12,0 |  |  |  | x |  |  |  | 13,0 |  |  | 11,0 |  |  |  | 17,0 |
|  | 200 | 10,0 | 10,0 | 10,0 | 20,0 | 10,0 |  | 10,0 |  | 9,0 | 14,0 |  |  | 9,0 |  | x |  |  | 18,0 |  |  | 13,0 |  |  |  | 19,0 |
|  | 277 |  |  |  |  |  |  |  |  | 12,0 |  |  |  |  |  |  | x |  |  |  |  |  |  |  |  | 12,3 |
| **EP 8** | 203 | 11,7 | 12,3 | 12,0 | 17,0 | 12,0 |  | 12,0 |  |  | 13,0 |  |  | 9,0 |  |  |  | x | 18,3 |  |  | 12,0 |  | 9,0 |  | 20,0 |
| **EP 10** | 67 |  |  |  |  |  |  |  |  |  |  |  |  |  |  |  |  |  | x |  |  |  |  |  |  |  |
|  | 208 | 10,0 |  | 9,0 |  | 9,0 |  |  |  |  |  |  |  |  |  |  |  |  |  | x |  |  |  |  |  | 19,3 |
|  | 210 | 9,0 |  |  |  | 9,0 |  |  |  |  |  |  |  |  |  |  |  |  |  |  | x |  |  |  |  | 21,0 |
| **EP 13** | 296,3 |  |  |  |  |  |  |  |  |  |  |  |  |  |  |  |  |  |  |  |  | x |  |  |  | 11,0 |
|  | 298,1 | 9,0 | 9,3 | 9,0 |  | 9,0 |  |  |  |  |  |  |  |  |  |  |  |  |  |  |  |  | x |  |  | 13,0 |
|  | 438 |  |  |  |  |  |  |  |  | 14,0 |  |  |  | 11,0 | 15,0 | 13,0 | 14,0 | 12,0 |  |  |  |  | 15,3 | x |  |  |
|  | 447 |  |  |  |  |  |  |  |  |  |  |  |  |  |  |  |  |  |  |  |  |  | 20,3 |  | x |  |
| **EP 14** | 508 |  |  |  |  |  |  |  |  |  |  |  |  |  |  |  |  |  |  |  |  |  |  |  |  | x |

^a^In the horizontal line the inoculated strains are given and in the vertical line the ones that were added as agar plug

**Table S5.** Grouping output of the complete dataset, 25 selected bacterial strains and the medium control

| Name | Samples Code | File | Similarity | Group 0.9 | Group 0.8 | Group 0.7 |
| --- | --- | --- | --- | --- | --- | --- |
| *Bacillus subtilis* EP4-170 | EP4-170 | TSRR0003_C-03_A1p_P4-C-3_01_54627.d | 0 | 1 | 1 | 1 |
| *Bacillus subtilis* EP4-170 | EP4-170 | TSRR0003_B-11_A1p_P4-B-11_01_54624.d | 0.989176 | 1 | 1 | 1 |
| *Bacillus subtilis* EP4-170 | EP4-170 | TSRR0003_B-12_A1p_P4-B-12_01_54625.d | 0.986345 | 1 | 1 | 1 |
| *Bacillus subtilis* EP7-199 | EP7-199 | TSRR0003_C-07_A1p_P4-C-7_01_54655.d | 0.974909 | 1 | 1 | 1 |
| *Bacillus subtilis* EP7-199 | EP7-199 | TSRR0003_C-09_A1p_P4-C-9_01_54657.d | 0.989418 | 1 | 1 | 1 |
| *Bacillus subtilis* EP7-199 | EP7-199 | TSRR0003_C-08_A1p_P4-C-8_01_54656.d | 0.985179 | 1 | 1 | 1 |
| *Bacillus subtilis* EP10-210 | EP10-210 | TSRR0003_D-09_A1p_P4-D-9_01_54666.d | 0.9628 | 1 | 1 | 1 |
| *Bacillus subtilis* EP10-210 | EP10-210 | TSRR0003_D-11_A1p_P4-D-11_01_54668.d | 0.995425 | 1 | 1 | 1 |
| *Bacillus subtilis* EP10-210 | EP10-210 | TSRR0003_D-10_A1p_P4-D-10_01_54667.d | 0.9902 | 1 | 1 | 1 |
| *Bacillus subtilis* EP7-200 | EP7-200 | TSRR0003_C-11_A1p_P4-C-11_01_54659.d | 0.973238 | 1 | 1 | 1 |
| *Bacillus subtilis* EP7-200 | EP7-200 | TSRR0003_C-10_A1p_P4-C-10_01_54658.d | 0.984451 | 1 | 1 | 1 |
| *Bacillus subtilis* EP7-200 | EP7-200 rep | TSRR0004_5_A1p_P1-B-5_01_55937.d | 0.931136 | 1 | 1 | 1 |
| *Bacillus subtilis* EP7-200 | EP7-200 rep | TSRR0004_6_A1p_P1-B-6_01_55938.d | 0.986411 | 1 | 1 | 1 |
| *Bacillus subtilis* EP7-200 | EP7-200 rep | TSRR0004_4_A1p_P1-B-4_01_55936.d | 0.975506 | 1 | 1 | 1 |
| *Bacillus* sp. EP5-815 | EP5-815 | TSRR0003_H-04_A1p_P4-H-4_01_54755.d | 0.909907 | 1 | 1 | 1 |
| *Bacillus* sp. EP5-815 | EP5-815 | TSRR0003_H-03_A1p_P4-H-3_01_54736.d | 0.992662 | 1 | 1 | 1 |
| *Bacillus* sp. EP6-195 | EP6-195 rep | TSRR0004_3_A1p_P1-B-3_01_55935.d | 0.898818 | 2 | 1 | 1 |
| *Bacillus* sp. EP6-195 | EP6-195 rep | TSRR0004_2_A1p_P1-B-2_01_55934.d | 0.996361 | 2 | 1 | 1 |
| *Bacillus* sp. EP6-195 | EP6-195 rep | TSRR0004_1_A1p_P1-B-1_01_55933.d | 0.974596 | 2 | 1 | 1 |
| *Bacillus* sp. EP7-277 | EP7-277 | TSRR0003_E-04_A1p_P4-E-4_01_54672.d | 0.791775 | 3 | 2 | 1 |
| *Bacillus* sp. EP7-277 | EP7-277 | TSRR0003_E-03_A1p_P4-E-3_01_54671.d | 0.990296 | 3 | 2 | 1 |
| *Bacillus* sp. EP7-277 | EP7-277 | TSRR0003_D-12_A1p_P4-D-12_01_54669.d | 0.966299 | 3 | 2 | 1 |
| *Bacillus* sp. EP6-454 | EP6-454 | TSRR0003_F-12_A1p_P4-F-12_01_54724.d | 0.95822 | 3 | 2 | 1 |
| *Bacillus* sp. EP6-454 | EP6-454 | TSRR0003_F-10_A1p_P4-F-10_01_54722.d | 0.997127 | 3 | 2 | 1 |
| *Bacillus* sp. EP6-454 | EP6-454 | TSRR0003_F-11_A1p_P4-F-11_01_54723.d | 0.996348 | 3 | 2 | 1 |
| *Bacillus* sp. EP6-817 | EP6-817 | TSRR0003_H-09_A1p_P4-H-9_01_54763.d | 0.848704 | 4 | 2 | 1 |
| *Bacillus* sp. EP6-817 | EP6-817 | TSRR0003_H-10_A1p_P4-H-10_01_54764.d | 0.970872 | 4 | 2 | 1 |
| *Bacillus* sp. EP5-815 | EP5-815 | TSRR0003_G-12_A1p_P4-G-12_01_54734.d | 0.935064 | 4 | 2 | 1 |
| *Bacillus* sp. EP6-817 | EP6-817 | TSRR0003_H-08_A1p_P4-H-8_01_54762.d | 0.941439 | 4 | 2 | 1 |
| *Bacillus sonorensis* EP13-298.1 | EP13-298.1 | TSRR0003_E-10_A1p_P4-E-10_01_54678.d | 0.699325 | 5 | 3 | 2 |
| *Bacillus sonorensis* EP13-298.1 | EP13-298.1 | TSRR0003_E-08_A1p_P4-E-8_01_54676.d | 0.980143 | 5 | 3 | 2 |
| *Bacillus sonorensis* EP13-298.1 | EP13-298.1 | TSRR0003_E-09_A1p_P4-E-9_01_54677.d | 0.968988 | 5 | 3 | 2 |
| *Bacillus* sp. EP6-195 | EP6-195 rep 2-3 | TSRR0005-12_A1p_P1-B-3_01_59098.d | 0.574131 | 6 | 4 | 3 |
| *Bacillus* sp. EP6-195 | EP6-195 rep 2-2 | TSRR0005-08_A1p_P1-A-8_01_59094.d | 0.963742 | 6 | 4 | 3 |
| *Bacillus* sp. EP6-195 | EP6-195 rep2-1 | TSRR0005-06_A1p_P1-A-6_01_59092.d | 0.945458 | 6 | 4 | 3 |
| *Bacillus* sp. EP6-195 | EP6-195 rep 2-2 | TSRR0005-07_A1p_P1-A-7_01_59093.d | 0.963381 | 6 | 4 | 3 |
| *Bacillus* sp. EP6-195 | EP6-195 rep2-1 | TSRR0005-04_A1p_P1-A-4_01_59090.d | 0.960878 | 6 | 4 | 3 |
| *Bacillus* sp. EP6-195 | EP6-195 rep 2-2 | TSRR0005-09_A1p_P1-A-9_01_59095.d | 0.934307 | 6 | 4 | 3 |
| *Bacillus* sp. EP6-195 | EP6-195 rep2-1 | TSRR0005-05_A1p_P1-A-5_01_59091.d | 0.987616 | 6 | 4 | 3 |
| *Bacillus* sp. EP6-195 | EP6-195 rep 2-3 | TSRR0005-10_A1p_P1-B-1_01_59096.d | 0.949749 | 6 | 4 | 3 |
| *Bacillus* sp. EP6-195 | EP6-195 rep 2-3 | TSRR0005-11_A1p_P1-B-2_01_59097.d | 0.976218 | 6 | 4 | 3 |
| *Bacillus* sp. EP8-203 | EP8-203 | TSRR0003_D-04_A1p_P4-D-4_01_54661.d | 0.84143 | 7 | 4 | 3 |
| *Bacillus* sp. EP8-203 | EP8-203 | TSRR0003_D-03_A1p_P4-D-3_01_54660.d | 0.989255 | 7 | 4 | 3 |
| *Bacillus* sp. EP8-203 | EP8-203 | TSRR0003_D-05_A1p_P4-D-5_01_54662.d | 0.972721 | 7 | 4 | 3 |
| *Bacillus* sp. EP1-654 | EP1-654 | TSRR0003_G-07_A1p_P4-G-7_01_54729.d | 0.853457 | 8 | 4 | 3 |
| *Bacillus* sp. EP1-654 | EP1-654 | TSRR0003_G-08_A1p_P4-G-8_01_54730.d | 0.942051 | 8 | 4 | 3 |
| *Bacillus* sp. EP1-654 | EP1-654 | TSRR0003_G-06_A1p_P4-G-6_01_54728.d | 0.848986 | 9 | 4 | 3 |
| *Bacillus* sp. EP13-296.3 | EP13-296.3 | TSRR0003_E-05_A1p_P4-E-5_01_54673.d | 0.616547 | 10 | 5 | 4 |
| *Bacillus* sp. EP13-296.3 | EP13-296.3 | TSRR0003_E-07_A1p_P4-E-7_01_54675.d | 0.990675 | 10 | 5 | 4 |
| *Bacillus* sp. EP13-296.3 | EP13-296.3 | TSRR0003_E-06_A1p_P4-E-6_01_54674.d | 0.982615 | 10 | 5 | 4 |
| *Bacillus stratosphericus* EP1-79 | EP1-79 | TSRR0003_B-05_A1p_P4-B-5_01_54618.d | 0.875983 | 11 | 5 | 4 |
| *Bacillus stratosphericus* EP1-79 | EP1-79 | TSRR0003_B-07_A1p_P4-B-7_01_54620.d | 0.988461 | 11 | 5 | 4 |
| *Bacillus stratosphericus* EP1-79 | EP1-79 | TSRR0003_B-06_A1p_P4-B-6_01_54619.d | 0.969844 | 11 | 5 | 4 |
| *Bacillus* sp. EP10-67 | EP10-67 | TSRR0003_B-04_A1p_P4-B-4_01_54617.d | 0.802926 | 12 | 5 | 4 |
| *Bacillus* sp. EP10-67 | EP10-67 | TSRR0003_A-12_A1p_P4-A-12_01_54615.d | 0.963492 | 12 | 5 | 4 |
| *Bacillus* sp. EP10-67 | EP10-67 | TSRR0003_B-03_A1p_P4-B-3_01_54616.d | 0.93112 | 12 | 5 | 4 |
| *Bacillus* sp. EP10-208 | EP10-208 | TSRR0003_D-06_A1p_P4-D-6_01_54663.d | 0.627381 | 13 | 6 | 5 |
| *Bacillus* sp. EP10-208 | EP10-208 | TSRR0003_D-07_A1p_P4-D-7_01_54664.d | 0.985177 | 13 | 6 | 5 |
| *Bacillus* sp. EP10-208 | EP10-208 | TSRR0003_D-08_A1p_P4-D-8_01_54665.d | 0.984266 | 13 | 6 | 5 |
| *Bacillus* sp. EP6-816 | EP6-816 rep | TSRR0004_9_A1p_P1-B-9_01_55941.d | 0.730369 | 14 | 7 | 5 |
| *Bacillus* sp. EP6-816 | EP6-816 rep | TSRR0004_8_A1p_P1-B-8_01_55940.d | 0.965884 | 14 | 7 | 5 |
| *Bacillus* sp. EP6-816 | EP6-816 rep | TSRR0004_7_A1p_P1-B-7_01_55939.d | 0.957094 | 14 | 7 | 5 |
| *Leclercia* sp. EP4-390 | EP4-390 | TSRR0003_E-12_A1p_P4-E-12_01_54680.d | 0.729582 | 15 | 8 | 5 |
| *Leclercia* sp. EP4-390 | EP4-390 | TSRR0003_F-03_A1p_P4-F-3_01_54681.d | 0.892871 | 16 | 8 | 5 |
| *Leclercia* sp. EP4-390 | EP4-390 | TSRR0003_E-11_A1p_P4-E-11_01_54679.d | 0.8741 | 17 | 8 | 5 |
| *Bacillus* sp. EP6-816 | EP6-816 | TSRR0003_H-05_A1p_P4-H-5_01_54756.d | 0.782622 | 18 | 9 | 5 |
| *Bacillus tequilensis* EP5-661 | EP5-661 | TSRR0003_G-09_A1p_P4-G-9_01_54731.d | 0.956815 | 18 | 9 | 5 |
| *Bacillus tequilensis* EP5-661 | EP5-661 | TSRR0003_G-10_A1p_P4-G-10_01_54732.d | 0.946889 | 18 | 9 | 5 |
| *Bacillus* sp. EP6-816 | EP6-816 rem | TSRR0003_H-07_rem_A1p_P4-H-7_01_55570.d | 0.900967 | 18 | 9 | 5 |
| *Bacillus* sp. EP6-816 | EP6-816 | TSRR0003_H-06_A1p_P4-H-6_01_54760.d | 0.876308 | 19 | 9 | 5 |
| *Bacillus tequilensis* EP5-661 | EP5-661 | TSRR0003_G-11_A1p_P4-G-11_01_54733.d | 0.79211 | 20 | 10 | 5 |
| *Pseudomonas balearica* EP13-447 | EP13-447 | TSRR0003_F-07_A1p_P4-F-7_01_54702.d | 0.611993 | 21 | 11 | 6 |
| *Pseudomonas balearica* EP13-447 | EP13-447 | TSRR0003_F-08_A1p_P4-F-8_01_54703.d | 0.970532 | 21 | 11 | 6 |
| *Bacillus* sp. EP13-438 | EP13-438 | TSRR0003_F-06_A1p_P4-F-6_01_54700.d | 0.912345 | 21 | 11 | 6 |
| *Bacillus* sp. EP13-438 | EP13-438 | TSRR0003_F-04_A1p_P4-F-4_01_54696.d | 0.849307 | 22 | 11 | 6 |
| *Bacillus* sp. EP13-438 | EP13-438 | TSRR0003_F-05_A1p_P4-F-5_01_54697.d | 0.897763 | 23 | 11 | 6 |
| *Pseudomonas balearica* EP13-447 | EP13-447 | TSRR0003_F-09_A1p_P4-F-9_01_54705.d | 0.808288 | 24 | 11 | 6 |
| *Bacillus thuringiensis* EP6-44 | EP6-44 | TSRR0003_A-10_A1p_P4-A-10_01_54613.d | 0.416388 | 25 | 12 | 7 |
| *Bacillus thuringiensis* EP6-44 | EP6-44 | TSRR0003_A-09_A1p_P4-A-9_01_54612.d | 0.995294 | 25 | 12 | 7 |
| *Bacillus thuringiensis* EP6-44 | EP6-44 | TSRR0003_A-11_A1p_P4-A-11_01_54614.d | 0.991094 | 25 | 12 | 7 |
| Medium Control | Medium Control rep2 | TSRR0005-03_A1p_P1-A-3_01_59089.d | 0.562337 | 26 | 13 | 8 |
| Medium Control | Medium Control rep2 | TSRR0005-02_A1p_P1-A-2_01_59088.d | 0.989265 | 26 | 13 | 8 |
| Medium Control | Medium Control rep2 | TSRR0005-01_A1p_P1-A-1_01_59087.d | 0.92888 | 26 | 13 | 8 |
| Medium Control | Medium Control | TSRR0003_A-05_A1p_P4-A-5_01_54608.d | 0.702332 | 27 | 14 | 8 |
| Medium Control | Medium Control | TSRR0003_A-04_A1p_P4-A-4_01_54607.d | 0.57114 | 28 | 15 | 9 |
| Medium Control | Medium Control | TSRR0003_A-03_A1p_P4-A-3_01_54606.d | 0.810841 | 29 | 15 | 9 |
| *Bacillus* sp. EP6-195 | EP6-195 | TSRR0003_C-06_A1p_P4-C-6_01_54654.d | 0.242599 | 30 | 16 | 10 |
| *Bacillus* sp. EP6-195 | EP6-195 | TSRR0003_C-05_A1p_P4-C-5_01_54653.d | 0.982618 | 30 | 16 | 10 |
| *Bacillus* sp. EP6-195 | EP6-195 | TSRR0003_C-04_A1p_P4-C-4_01_54628.d | 0.978819 | 30 | 16 | 10 |
| *Lysinibacillus* sp. EP1-81 | EP1-81 | TSRR0003_B-10_A1p_P4-B-10_01_54623.d | 0.888261 | 31 | 16 | 10 |
| *Lysinibacillus* sp. EP1-81 | EP1-81 | TSRR0003_B-09_A1p_P4-B-9_01_54622.d | 0.962261 | 31 | 16 | 10 |
| *Lysinibacillus* sp. EP1-81 | EP1-81 | TSRR0003_B-08_A1p_P4-B-8_01_54621.d | 0.886927 | 32 | 16 | 10 |
| *Bacillus* sp. EP1-11 | EP1-11 | TSRR0003_A-07_A1p_P4-A-7_01_54610.d | 0.693656 | 33 | 17 | 11 |
| *Bacillus* sp. EP1-11 | EP1-11 | TSRR0003_A-08_A1p_P4-A-8_01_54611.d | 0.912174 | 33 | 17 | 11 |
| *Bacillus* sp. EP1-11 | EP1-11 | TSRR0003_A-06_A1p_P4-A-6_01_54609.d | 0.875067 | 34 | 17 | 11 |
| *Bacillus* sp. EP6-816 | EP6-816 | TSRR0003_H-07_A1p_P4-H-7_01_54761.d | 0.179614 | 35 | 18 | 12 |
| *Brevibacterium* sp. EP14-508 | EP14-508 | TSRR0003_G-05_A1p_P4-G-5_01_54727.d | 0.074954 | 36 | 19 | 13 |
| *Brevibacterium* sp. EP14-508 | EP14-508 | TSRR0003_G-03_A1p_P4-G-3_01_54725.d | 0.854807 | 37 | 19 | 13 |
| *Brevibacterium* sp. EP14-508 | EP14-508 | TSRR0003_G-04_A1p_P4-G-4_01_54726.d | 0.822315 | 38 | 19 | 13 |

Strains are indicated by their internal identifier. Metabolic groups according to the chemical diversity are given for a similarity threshold of 0.9, 0.8 and 0.7, respectively

**Table S6.** Inspected metabolic groups (n=3)

| **Strain** | **Metabolic group** |
| --- | --- |
| EP4-170 | 1 |
| EP7-199 | 1 |
| EP10-210 | 1 |
| EP7-200 rep | 1 |
| EP5-815 | 1 |
| EP7-277 | 2 |
| EP6-454 | 2 |
| EP6-817 | 3 |
| EP13-298.1 | 4 |
| EP6-195 rep2-1 | 5 |
| EP8-203 | 6 |
| EP1-654 | 7 |
| EP13-296.3 | 8 |
| EP1-79 | 9 |
| EP10-67 | 10 |
| EP10-208 | 11 |
| EP6-816 rep | 12 |
| EP4-390 | 13 |
| EP6-816 | 14 |
| EP5-661 | 14 |
| EP13-447 | 15 |
| EP13-438 | 15 |
| EP6-44 | 16 |
| Medium Control |  |
| EP1-81 | 17 |
| EP1-11 | 18 |
| EP14-508 | 19 |

**Table S7.** Antibacterial activity of selected strains against Gram-negative (*E. coli*) and Gram-positive bacteria (*M. luteus*) in 10 different media

| **No** | **Strain** | **200** | | | | **203** | | | | **654** | | | | **815** | | | | **816** | | | |
| --- | --- | --- | --- | --- | --- | --- | --- | --- | --- | --- | --- | --- | --- | --- | --- | --- | --- | --- | --- | --- | --- |
|  |  | **1** | | **2** | | **1** | | **2** | | **1** | | **2** | | **1** | | **2** | | **1** | | **2** | |
|  | **Media** | **Ec** | **Ml** | **Ec** | **Ml** | **Ec** | **Ml** | **Ec** | **Ml** | **Ec** | **Ml** | **Ec** | **Ml** | **Ec** | **Ml** | **Ec** | **Ml** | **Ec** | **Ml** | **Ec** | **Ml** |
| 1 | Flam+Asw | NT | NT | - | +++ | NT | NT | - | ++ | NT | NT | - | - | NT | NT | - | +++ | NT | NT | - | +++ |
| 2 | ISP2+NaCl | +++ | ++ | - | +++ | - | +++ | - | +++ | NT | NT | - | +++ | NT | NT | - | + | NT | NT | - | + |
| 3 | LB | NT | NT | - | +++ | NT | NT | - | +++ | NT | NT | - | + | NT | NT | + | +++ | NT | NT | - | +++ |
| 4 | LB+ASW | NT | NT | - | 16 | NT | NT | - | +++ | NT | NT | - | ++ | NT | NT | - | +++ | NT | NT | - | +++ |
| 5 | MB | NT | NT | - | - | NT | NT | - | +++ | NT | NT | - | - | NT | NT | - | + | NT | NT | - | + |
| 6 | MYE | NT | NT | - | ++ | NT | NT | - | +++ | NT | NT | +++ | ++ | NT | NT | - | ++ | NT | NT | - | + |
| 7 | NA | NT | NT | - | ++ | NT | NT | - | ++ | - | ++++ | - | ++ | - | ++ | + | +++ | - | +++ | - | +++ |
| 8 | SNA+ASW | NT | NT | - | + | NT | NT | - | - | NT | NT | - | - | NT | NT | - | + | NT | NT | - | ++ |
| 9 | TSA | NT | NT | - | +++ | NT | NT | - | ++ | NT | NT | - | - | NT | NT | - | +++ | NT | NT | - | ++ |
| 10 | ISP2 | NT | NT | + | +++ | NT | NT | ++ | +++ | NT | NT | - | + | NT | NT | - | ++ | NT | NT | - | ++ |

1 : First screening + : weak activity - : no activity Ml : *Micrococcus luteus*

2 : Second screening ++ : moderate activity NT : Not tested Ec : *Escherichia coli*

+++ : strong activity

**Table S8.** MIC values of surfactins and macrolactin A (all MIC values were obtained from three independent measurements, n = 3).

| **Tested cpd** | **MIC (µg/mL)** | | | | | |
| --- | --- | --- | --- | --- | --- | --- |
|  | ***E. coli*** | ***E. coli*** | ***B. subtilis*** | ***S. aureus*** | ***S. aureus*** | ***L. monocytogenes*** |
|  | **ATCC 25922** | **ATCC 25922** | **DSM 10** | **ATCC 25923** | **ATCC 33592** | **DSM 20600** |
|  | **wild type** | **ΔTolC** | **wild type** | **MSSA** | **MRSA** | **wild type** |
| 1: C14 surfactin (n=3) | > 128 | > 128 | > 128 | > 128 | > 128 | > 128 |
| 2: C15 surfactin (n=3) | > 128 | > 128 | > 128 | > 128 | > 128 | > 128 |
| 3: C16 surfactin (n=3) | > 128 | > 128 | > 128 | > 128 | > 128 | > 128 |
| 4: C17surfactin (n=3) | > 128 | > 128 | > 128 | > 128 | > 128 | > 128 |
| 5: Macrolactin A (n=3) | > 128 | > 128 | > 128 | 4-8 | 2-4 | 2-32 |
| Gentamycin control (n=3) | 0.25-1 | 0.125-0.5 | ≤ 0.063 | 0.125 | 0.125-0.25 | 0.063 |

**Table S9.** Combination effect between macrolactin A and C14 surfactin

| **Cpd** | ***E. coli* ATCC25922** | ***B. subtilis* DSM10** | ***S. aureus* ATCC33592(MRSA)** |
| --- | --- | --- | --- |
| C14 surfactin | Not active | Not active | Not active |
| Macrolactin A | Not active | Not active | Active |
| Combination | No effect | No effect | Synergistic effect |

**References**

1. Hooper, J. N. A. & Van Soest, R. W. M. Systema porifera. A guide to the classification of sponges. in *Hooper J.N.A., Van Soest R.W.M., Willenz P. (eds) Systema Porifera* (Springer US, 2002).

2. Van Soest, R. W. M., Erpenbeck, D. & Alvarez, B. Family Dictyonellidae Van Soest, Diaz & Pomponi, 1990. *Syst. Porifera* 773–786 (2002) doi:10.1007/978-1-4615-0747-5_83.

3. *Stylissa flabelliformis* – Sponges of Polynesia. https://sponges-tahiti.ird.fr/?p=899.

4. Subagio, I. B., Setiawan, E., Hariyanto, S. & Irawan, B. Spicule size variation in *Xestospongia testudinaria* Lamarck, 1815 at Probolinggo-Situbondo coastal. *AIP Conf. Proc.* **1854**, (2017).

5. Pawlik, J. R., McFall, G. & Zea, S. Does the odor from sponges of the genus *Ircinia* protect them from fish predators? *J. Chem. Ecol.* **28**, 1103–1115 (2002).

6. Wiedenmayer, F. *Shallow-water sponges of the western Bahamas*. *Experientia: Suppl.;28* vol. 1 (1977).

7. Keratosa, P. I. & Soest, R. Van. Marine sponges from Curaçao and other Caribbean. *Stud. Fauna Curaçao other Caribb. Islands* **56**, 1–94 (1978).

8. Lukowiak, M., Piser, A. & O’dea, A. Do spicules in sediments reflect the living sponge community? A test in a Caribbean shallow-water lagoon. *Palaios* **28**, 373–385 (2013).

9. Neopetrosia carbonaria. https://guide.poriferatreeoflife.org/sp_38.html.

10. Vicente, J., Ríos, J. A., Zea, S. & Toonen, R. J. Molecular and morphological congruence of three new cryptic *Neopetrosia* spp. in the Caribbean. *PeerJ* **7**, e6371 (2019).

11. Dewi, A. S., Hadi, T. A., Januar, H. I., Pratitis, A. & Chasanah, E. Study on the effect of pollutants on the production of aaptamines and the cytotoxicity of crude extract from *Aaptos suberitoides*. *Squalen Bull. Mar. Fish. Postharvest Biotechnol.* **7**, 97 (2013).

12. Calcinai, B. *et al.* Demosponge diversity from North Sulawesi, with the description of six new species. *Zookeys* **680**, 105–150 (2017).

13. Dewi, A. S. *et al.* Acanthocyclamine A From the Indonesian Marine Sponge *Acanthostrongylophora ingens*. *Aust. J. Chem.* **67**, 1205 (2014).

14. Lévi, C. Sponges of the New Caledonian Lagoon. *Editions de l’Orstom, Institut Francais de Recherche Scientifique pour le Development en Cooperation, Collection Faune et flore tropicales n° XXXIII, Paris (1998).*

15. Rohde, S. & Schupp, P. J. Allocation of chemical and structural defenses in the sponge *Melophlus sarasinorum*. *J. Exp. Mar. Bio. Ecol.* **399**, 76–83 (2011).

16. Hoshino, T. Description of two new species in the genus *Agelas* (Demospongia) from Zamami Island, the Ryukyus, Japan. *Jap.Soc.Syst. Zool* 1–10 (1985).
